# Cardiomyocyte GSDME Drives Doxorubicin-Induced Cardiotoxicity by Targeting the CCL2-CCR2 Axis

**DOI:** 10.7150/ijbs.115489

**Published:** 2025-09-29

**Authors:** Junqiang Xue, Shiyao Xie, Xuelin Cheng, Enyong Su, Xiaoyue Song, Lili Wei, Peng Yu, Ming Liu, Hong Jiang

**Affiliations:** 1 Department of Cardiology, Zhongshan Hospital, Fudan University, Shanghai Institute of Cardiovascular Diseases, Shanghai, 200032, China.; 2 Department of Cardiology, the First Affiliated Hospital of Zhengzhou University, Zhengzhou, 450000, China.; 3 Department of General Practice, Zhongshan Hospital, Fudan University, Shanghai, 200032, China.; 4 Shanghai Engineering Research Center of AI Technology for Cardiopulmonary Diseases, Zhongshan Hospital, Fudan University, Shanghai, 200032, China.; 5 Shanghai Xuhui Central Hospital, Zhongshan-Xuhui Hospital, Fudan University, Shanghai, 200032, China.; 6 Department of Endocrinology and Metabolism, Shanghai Geriatric Medical Center, Shanghai, 200032, China.; 7 Department of Health Management Center, Zhongshan Hospital, Fudan University, Shanghai, 200032, China.; 8 Innovative Center for New Drug Development of Immune Inflammatory Diseases, Ministry of Education, Fudan University, Shanghai, 200032, China.; 9 State Key Laboratory of Cardiovascular Diseases, Zhongshan Hospital, Fudan University, Shanghai, 200032, China.; 10 NHC Key Laboratory of Ischemic Heart Diseases, Shanghai, 200032, China.; 11 Key Laboratory of Viral Heart Diseases, Chinese Academy of Medical Sciences, Shanghai, 200032, China.; 12 National Clinical Research Center for Interventional Medicine, Shanghai, 200032, China.

**Keywords:** Gasdermin E, CCL2-CCR2 axis, STING/NFκB Signaling, Doxorubicin-Induced Cardiotoxicity.

## Abstract

The use of doxorubicin (DOX), a wide-spectrum antineoplastic drug, is largely limited in clinical practice because of the less than satisfactory strategies available for the prevention and treatment of doxorubicin-induced cardiotoxicity (DIC). Although gasdermin E (GSDME) has been linked to the development of several cardiovascular diseases, the role of GSDME in DIC has not been thoroughly investigated. Here, we aimed to explore the role of GSDME in the development of DIC and develop efficient and feasible targets for managing DIC. We found that GSDME was upregulated in both DOX-treated murine hearts and isolated adult mouse cardiomyocytes. Conditional *Gsdme* knockout in cardiomyocytes, but not myeloid cells, attenuated DOX-induced cardiac remodeling, cardiac malfunction, and cardiac fibrosis. Cardiomyocyte-specific *Gsdme* knockout dampened DOX-induced cardiac CCL2-CCR2 signaling and inflammation, while CCL2 inhibition or CCR2+ macrophage depletion attenuated DIC. Mechanistically, GSDME facilitated mitochondrial injury in cardiomyocytes to release mtDNA and activated the STING/NFκB pathway, further targeting the CCL2-CCR2 axis and cardiac inflammation, thereby aggravating DIC. These findings identify GSDME as a potential therapeutic target for DIC.

## Introduction

Doxorubicin (DOX), a representative drug in the class of anthracycline compounds, is widely prescribed in chemotherapy regimens for a diverse range of hematological malignancies and solid tumors^1^, ^2^. Although it is used extensively and has high therapeutic efficacy, DOX is also the main cause of chemotherapy-induced cardiotoxicity^3^, ^4^. To date, dexrazoxane is the only drug approved by the US Food and Drug Administration to attenuate doxorubicin-induced cardiotoxicity (DIC). However, dexrazoxane can reduce sensitivity to antitumor therapy and increase the risk of secondary malignancies, and its use has been strictly limited to only a small proportion of potentially eligible patients^5^. Elucidating the underlying mechanisms of DIC is urgently needed to develop feasible and effective strategies for preventing and treating this complication.

There is growing evidence that cardiac inflammation is an important mechanism of DIC pathogenesis^6^-^9^. Among other pathways, the C-C motif chemokine ligand 2 (CCL2)-C-C motif chemokine receptor 2 (CCR2) axis plays important roles in a variety of cardiovascular diseases, including heart failure^10^, atherosclerosis^11^, myocardial infarction^12^, ^13^, hypertension^14^, and myocarditis^15^. In addition, higher circulating CCL2 levels have been associated with long-term cardiovascular mortality in community-dwelling individuals free of overt cardiovascular disease^16^. CCL2, also known as monocyte chemoattractant protein-1 (MCP-1), is one of the earliest discovered and most functionally well-defined proteins of the CC family of chemokines^17^. CCL2 is secreted by a variety of cells, including immune cells, smooth muscle cells, endothelial cells, cystoid cells, and fibroblasts^17^-^19^. In contrast, CCR2 expression is restricted mainly to bone marrow, hematopoietic cells, and secondary lymphoid organs, and CCR2 is expressed predominantly by monocytes^20^. In DIC, monocyte-derived macrophages in the heart mainly exhibit a proinflammatory phenotype^21^. As an important class of cardiac macrophages, CCR2+ macrophages enter the heart during the first week of life and are renewed through the recruitment of circulating monocytes and self-proliferation. Although the function of CCR2+ macrophages has not been fully elucidated, it is widely recognized that CCR2+ macrophages have proinflammatory potential that can trigger cardiac inflammation and ultimately lead to heart failure^13^, ^22^. Several studies have shown that CCL2 expression is elevated in DOX-stimulated cardiac tissue and cardiomyocytes^23^-^25^. However, the role of the CCL2-CCR2 axis in DIC has not been well studied.

Gasdermins are a family of proteins characterized by their ability to form pores and are primarily involved in the processes of pyroptosis and inflammation. The members of this family identified to date include GSDMA, GSDMB, GSDMC, GSDMD, GSDME (also known as DFNA5), and PJVK^26^-^28^. Except for PJVK, gasdermins possess a core pore-forming N-terminal domain that is self-inactivated by its C-terminal domain in the quiescent state. After proteolytic cleavage, the released N-terminal fragments are inserted into and thus perforate the cellular membrane, disrupting organelles or the plasma membrane and causing the leakage of intracellular contents. The first identified gasdermin protein, GSDMD, which is activated mainly by caspase 1/11, has been reported to promote the pathological development of various cardiovascular diseases^29^-^35^. Another gasdermin protein, GSDME, is cleaved by caspase 3 and promotes the transition from noninflammatory apoptosis to pyroptosis^36^. Accumulating evidence has shown that chemotherapy and radiation act on GSDME to suppress tumor development^37^-^39^. However, unlike GSDMD, GSDME is silenced in most cancer cells but widely expressed in normal tissues, suggesting that GSDME functions as more than a tumor suppressor^36^. Recent research has shown that GSDME is involved in the development of atherosclerosis and immune checkpoint inhibitor-associated myocarditis^40^, ^41^. Several studies have demonstrated the rudimentary role of GSDME in DIC^42^-^44^.

In this study, we systematically investigated the role of GSDME both *in vivo* and* in vitro*. Our findings revealed that global or conditional *Gsdme* knockout in cardiomyocytes but not in myeloid cells attenuated DIC. Moreover, cardiomyocyte-conditional *Gsdme* knockout decreased CCL2-CCR2 signaling and inflammatory response, whereas CCL2 inhibition with bindarit or CCR2+ macrophage depletion exerted a remarkable cardioprotective effect on DIC. Mechanistically, cardiomyocyte GSDME targeted mitochondrial damage-induced mtDNA leakage, further activating the STING (stimulator of interferon genes)/NFκB (nuclear factor kappa B) pathway and promoting CCL2-CCR2 signaling and cardiac inflammation, culminating in the development of DIC. We identified GSDME as a crucial driver of DIC and suggest that targeting GSDME may provide a novel strategy for the prevention and treatment of this condition.

## Materials and Methods

The expanded Methods section is available in the supplementary materials online.

### Animal experiments

To establish a chronic DIC animal model, eight-week-old mice were administered DOX at a weekly dose of 5 mg/kg via intraperitoneal injection for 4 consecutive weeks for a cumulative dose of 20 mg/kg.

Standard protocols for histology, echography, molecular biological analysis, cytokine array analysis, and flow cytometry were employed, as detailed in the Methods section in the supplementary materials online.

### Cell treatment

Adult mouse cardiomyocytes (AMCMs) were isolated using a Langendorff-free method as previously described^45^. To explore the role of DOX in cardiomyocytes, 1 μM DOX was added to cultured AMCMs. Standard protocols were employed for cell fractionation, evaluation of mitochondrial damage, and molecular biological analysis, as detailed in the Methods section in the supplementary materials online.

### Statistical analysis

All data are presented as the mean±SEM. Statistical analyses were carried out in GraphPad Prism 10.2.2 software.

## Results

### Global* Gsdme* knockout prevents DIC

**Figure 1:**
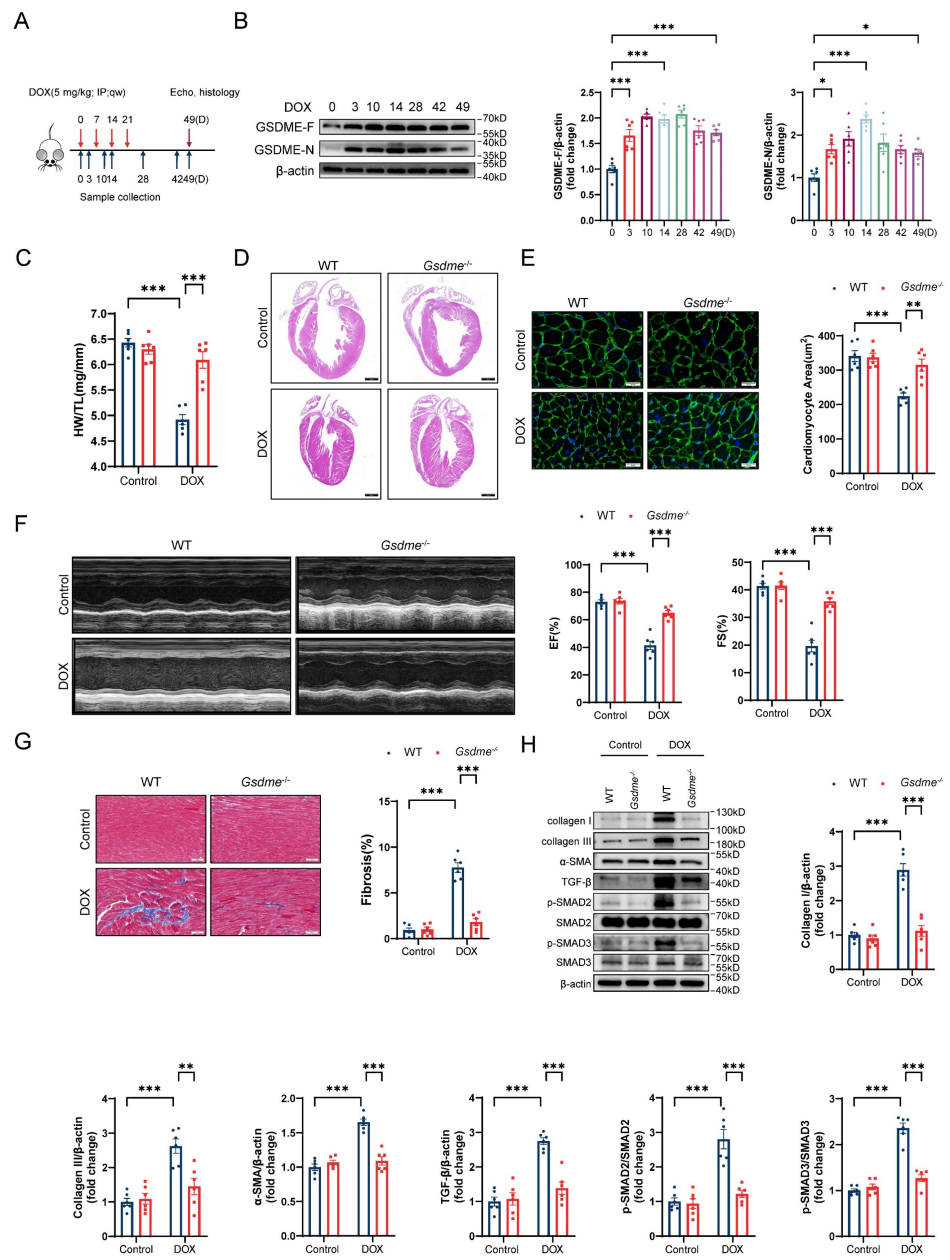
** Global *Gsdme* knockout prevents DIC. A,** Diagram of the experimental design for the establishment of a chronic doxorubicin-induced cardiotoxicity (DIC) mouse model. **B,** Western blot and quantitative analysis of full-length GSDME (GSDME-F) and cleaved N-terminal GSDME (GSDME-N) in the hearts of DOX-treated WT mice at the indicated times (days 0, 3, 10, 14, 28, 42, and 49); n = 6 per group. **C,** heart weight (HW) normalized to tibia length (TL); n = 6 per group. **D,** Representative hematoxylin and eosin (H&E) staining of WT or *Gsdme^-/-^* murine hearts on day 49 after DOX treatment. Scale bar = 1000 μm. **E,** Representative wheat germ agglutinin (WGA) staining and quantification of the cardiomyocyte cross-sectional area of WT or *Gsdme^-/-^* murine hearts on day 49 after DOX treatment. Scale bar = 20 μm; n = 6 per group. **F,** Representative left ventricular M-mode images and echocardiographic measurements of the ejection fraction (EF) and fractional shortening (FS) of WT or *Gsdme^-/-^* mice on day 49 after DOX treatment; n = 6 per group. **G,** Representative Masson's trichrome staining and relative quantification of the myocardial fibrosis area in WT or *Gsdme^-/-^* murine hearts on day 49 after DOX treatment. Scale bar = 50 μm; n = 6 per group. **H,** Representative immunoblot images and densitometric analysis of collagen I, collagen III, α-SMA, and TGF-β/SMAD2/SMAD3 pathway-associated proteins in heart tissues from WT or *Gsdme^-/-^* mice on day 49 after DOX treatment; n = 6 per group. *P < 0.05, **P < 0.01, ***P < 0.001.

To explore the role of GSDME in DIC, we developed a mouse model of chronic DIC, as shown in Figure 1A. Eight-week-old C57BL/6J mice received a cumulative dose of 20 mg/kg DOX via intraperitoneal injection at a weekly dosage of 5 mg/kg for 4 consecutive weeks as previously described^46^. The mice were sacrificed at the indicated times, and the hearts were harvested for the evaluation of GSDME levels. The levels of both full-length GSDME and cleaved N-terminal GSDME were increased on days 3, 10, 14, 28, 42, and 49 following DOX administration and peaked around day 14 (Figure 1B). We subsequently developed a global *Gsdme* knockout mouse strain (*Gsdme^-/-^*) using the CRISPR/Cas9 strategy (Figure S1A). Genotyping and immunoblotting confirmed the ablation of GSDME in murine hearts (Figure S1B and S1C). Compared with control mice, DOX-treated wild-type (WT) mice had reduced heart weights, heart sizes, and cardiomyocyte cross-sectional areas. However, these atrophic cardiac alterations were mitigated by global *Gsdme* knockout (Figure 1C through 1E). We used echocardiography to evaluate cardiac function after DOX treatment. Significant decreases in the left ventricular ejection fraction (EF) and in fractional shortening (FS) were observed in DOX-treated WT mice but significantly reversed in DOX-treated *Gsdme^-/-^* mice (Figure 1F). After DOX treatment in WT mice, Masson's trichrome staining revealed an increase in the fibrotic area, and immunoblot analysis demonstrated the upregulation of fibrosis-related proteins, including collagen I, III, and α-SMA, together indicating myocardial fibrosis. However, these changes were blunted in *Gsdme^-/-^* mice (Figure 1G and 1H). TGF-β/SMAD2/SMAD3 signaling dominates cardiac fibrosis^47^. Compared with DOX-treated WT mice, DOX-treated *Gsdme^-/-^* mice exhibited inactivation of TGF-β/SMAD2/SMAD3 signaling (Figure 1H). Taken together, these results indicate that global *Gsdme* knockout prevented the development of the detrimental phenotypes of DIC.

### *Gsdme* knockout in cardiomyocytes, but not in myeloid cells, prevents DIC

**Figure 2:**
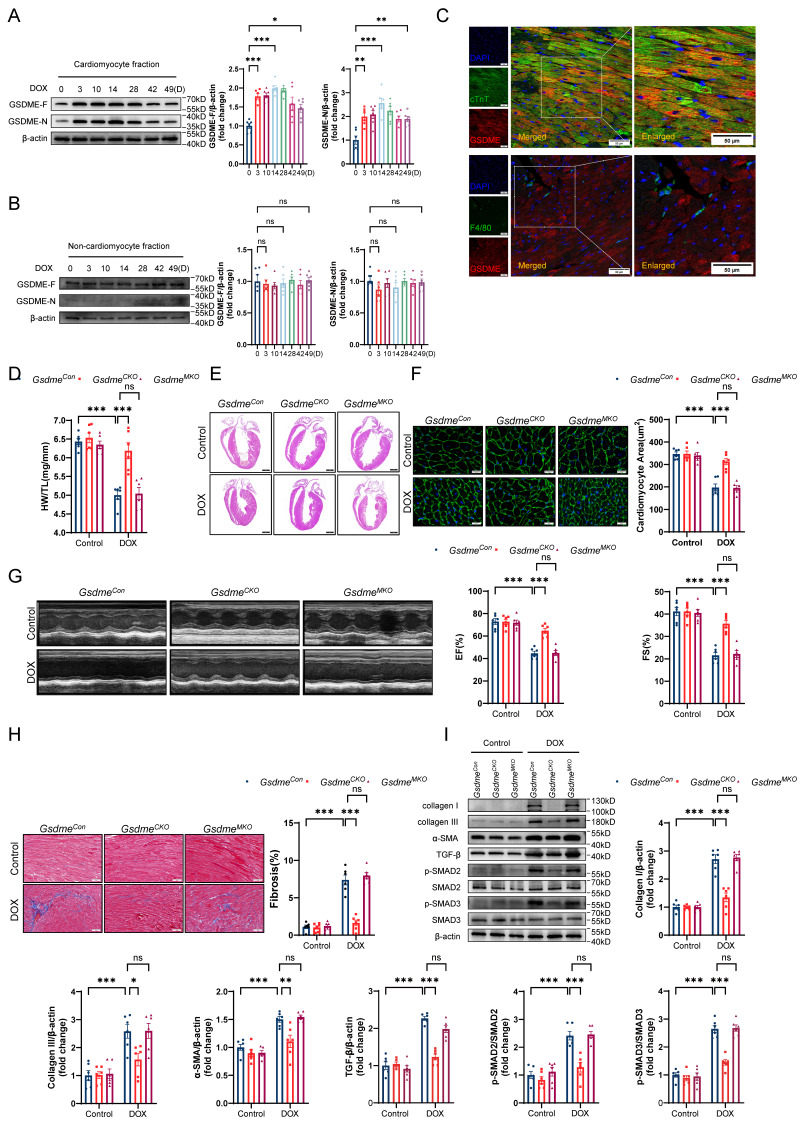
***Gsdme* knockout in cardiomyocytes, but not in myeloid cells, prevents DIC. A and B,** Western blot and relative quantification of full-length GSDME and cleaved N-terminal GSDME in isolated adult mouse cardiomyocyte (AMCM) fraction **A,** and in cardiac noncardiomyocyte fraction **B,** from DOX-treated WT mice; n = 6 per group. **C,** Representative fluorescence staining of GSDME (red), cTnT (green), and F4/80 (green) in mouse hearts on day 14 after DOX treatment. Insets indicate an enlarged view. Scale bar = 50 μm. **D,** Ratio of HW to TL; n = 6 per group. **E,** Representative H&E staining of hearts from *Gsdme^Con^*, *Gsdme^CKO^*, and *Gsdme^MKO^* mice after DOX administration. Scale bar = 1000 μm. **F,** WGA staining and measurements of cardiomyocyte cross-sectional area in heart sections from DOX-treated *Gsdme^Con^, Gsdme^CKO^*, and *Gsdme^MKO^* mice. Scale bar = 20 μm; n = 6 per group. **G,** Representative echocardiographic images and quantitative analysis of cardiac contractile function, including left ventricular EF and FS, in *Gsdme^Con^*, *Gsdme^CKO^*, and *Gsdme^MKO^* mice receiving DOX therapy; n = 6 per group. **H,** Masson's trichrome staining and quantification of cardiac fibrosis after DOX treatment on *Gsdme^Con^, Gsdme^CKO^*, and *Gsdme^MKO^* mice. Scale bar = 50 μm; n = 6 per group. **I,** Representative immunoblot images and quantitative histograms of collagen I, collagen III, α-SMA, TGF-β, phospho-SMAD2, total-SMAD2, phospho-SMAD3, and total-SMAD3 in hearts from *Gsdme^Con^, Gsdme^CKO^*, and *Gsdme^MKO^* mice exposed to DOX; n = 6 per group. ns indicated no significance, *P < 0.05, **P < 0.01, ***P < 0.001.

To clarify the specific cardiac cell types in which GSDME promotes DIC, we isolated cardiomyocytes and noncardiomyocytes from the hearts of mice exposed to DOX at the indicated times. Consistent with the observations in murine hearts, full-length GSDME and cleaved N-terminal GSDME in the isolated adult mouse cardiomyocyte (AMCM) fraction were upregulated on day 3 and persisted until day 49 in the DIC model (Figure 2A), whereas no such changes were observed in the noncardiomyocyte fraction (Figure 2B). We then performed double immunofluorescence staining and found that GSDME was located mainly in cTnT-positive cardiomyocytes, with a negligible signal in F4/80-positive macrophages (Figure 2C). *In vitro* experiments revealed that total and cleaved GSDME in WT AMCMs were increased significantly at 6, 12, and 24 hours after stimulation with 1 μM DOX, whereas GSDME was almost undetectable in *Gsdme^-/-^* AMCMs (*Gsdme* KO) (Figure S2A). Given that chemotherapy classically activates caspase-3, which is upstream of GSDME cleavage^36^, we investigated whether GSDME cleavage in cardiomyocytes is indeed dependent on caspase-3. We observed activated caspase-3 in parallel with the cleavage of GSDME after doxorubicin exposure (Figure S2B). We subsequently introduced the caspase-3 inhibitor zVAD-FMK to doxorubicin-induced cardiomyocytes and found that treatment with zVAD-FMK blocked doxorubicin-induced GSDME processing into its N-terminal fragment, suggesting that GSDME activation in cardiomyocytes is dependent on caspase-3 (Figure S2B). These findings suggest that GSDME was primarily expressed and activated in cardiomyocytes in our DIC model.

Next, we generated a mouse strain carrying loxP recombination sites flanking the *Gsdme* gene (*Gsdme^f/f^*) (Figure S2C). Conditional *Gsdme* knockout in cardiomyocytes (*Gsdme^f/f^Myl2*^Cre*/+*^*, Gsdme^CKO^*) or in myeloid cells (*Gsdme^f/f^Lyz2*^Cre*/+*^*, Gsdme^MKO^*) was achieved by crossing *Gsdme^f/f^* mice with *Myl2-*Cre or *Lyz2-*Cre transgenic mice (Figure S2D and S2E). Immunoblotting confirmed the marked decrease in GSDME in the hearts of *Gsdme^CKO^* mice and in the bone marrow-derived macrophages of *Gsdme^MKO^* mice (Figure S2F and S2G). After DOX treatment, *Gsdme^CKO^* mice but not *Gsdme^MKO^* mice exhibited preserved heart weights, heart sizes, and cardiomyocyte sizes compared with littermate-negative mice (*Gsdme^Con^*) (Figure 2D through 2F). Echocardiography revealed that the DOX-induced reduction in EF and FS in *Gsdme^Con^* mice was inhibited in *Gsdme^CKO^* mice but not in *Gsdme^MKO^* mice (Figure 2G). *Gsdme* knockout in cardiomyocytes but not in myeloid cells inhibited myocardial fibrosis induced by DOX (Figure 2H). Both DOX-induced upregulation of collagen I, III, and α-SMA expression and activation of the TGF-β/SMAD2/SMAD3 pathway in the hearts of *Gsdme^Con^* mice were also significantly inhibited by *Gsdme* knockout in cardiomyocytes but not in myeloid cells (Figure 2I). Taken together, these findings indicate an indispensable role for GSDME in cardiomyocytes in DIC.

### *Gsdme* knockout reduces CCL2-CCR2 signaling and cardiac inflammation

**Figure 3:**
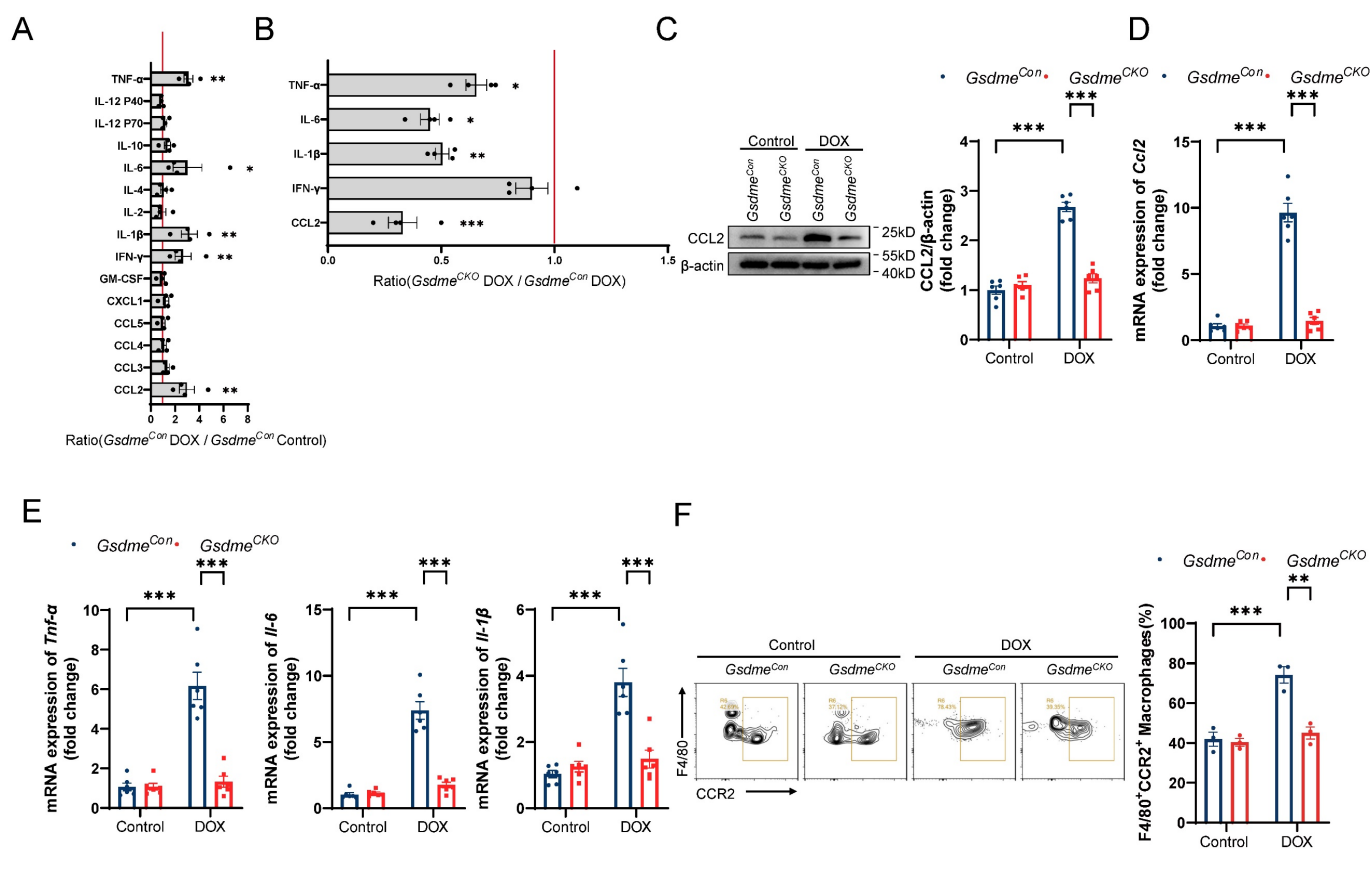
***Gsdme* knockout reduces CCL2-CCR2 signaling and cardiac inflammation. A,** Cytokine array evaluation of the levels of inflammatory factors in the hearts of *Gsdme^Con^* mice treated with DOX; n = 4 per group. **B,** Levels of five inflammatory factors in the hearts of *Gsdme^CKO^* mice compared with those in the hearts of *Gsdme^Con^* mice after DOX treatment; n = 4 per group. **C,** Representative immunoblotting and relative quantitative analysis of CCL2 expression in mouse hearts; n = 6 per group. **D and E,** RT‒qPCR analysis of the transcription levels of *Ccl2* **D)**, *Tnf-α*, *Il-6*, and *Il-1β* **E)** in mouse hearts; n = 6 per group. **F,** Representative flow cytometry images and quantitative analysis of mouse heart CD45+CD11b+F4/80+CCR2+ macrophages; n = 3 per group. * p < 0.05, ** p < 0.01, *** p < 0.001.

Zhang *et al.* reported significant increases in the levels of cardiac inflammatory factors and the numbers of inflammatory cells at the early stage of DOX administration^21^; therefore, we used a cytokine array to detect changes in representative inflammatory factors in the hearts of mice on day 14 of DIC modeling. Changes in cytokine levels in the hearts of DOX-treated *Gsdme^Con^* mice compared with those in the hearts of saline-treated *Gsdme^Con^* mice are shown in Figure 3A. Among them, the levels of CCL2, IFN-γ, IL-1β, IL-6, and TNF-α were significantly increased after DOX administration. Among the 5 cytokines/chemokines whose expression tended to increase in the *Gsdme^Con^* mice, CCL2 was decreased more in *Gsdme^CKO^* mice than in *Gsdme^Con^* mice (Figure 3B). Both immunoblotting and RT‒qPCR revealed that the level of CCL2 increased after DOX treatment but was inhibited by *Gsdme* knockout (Figure 3C and 3D). RT‒qPCR also revealed that cardiomyocyte-specific *Gsdme* knockout reduced the levels of cardiac inflammatory markers including *Tnf-α, Il-6,* and* Il-1β* (Figure 3E)*.* CCR2 is the predominant receptor for CCL2. We then isolated immune cells from the hearts of *Gsdme^Con^* mice and *Gsdme^CKO^* mice on day 14 after DOX administration and further stained them with anti-CD45, anti-CD11b, anti-F4/80, and anti-CCR2 antibodies for multiplexed flow cytometry. We determined the effect of cardiomyocyte-specific *Gsdme* knockout on cardiac CCR2+ macrophages by manual gating (Figure S3). The flow cytometry results revealed that DOX treatment significantly increased the percentage of CD45+CD11b+F4/80+CCR2+ macrophages in the hearts of *Gsdme^Con^* mice (Figure 3F). However, cardiomyocyte-conditional *Gsdme* knockout inhibited the upregulation of CD45+CD11b+F4/80+CCR2+ macrophages in DOX-induced cardiac tissue (Figure 3F). These results indicate that cardiomyocyte-conditional *Gsdme* knockout inhibited cardiac CCL2-CCR2 axis activity and inflammation in DIC model mice.

### The CCL2 inhibitor bindarit prevents DIC

**Figure 4:**
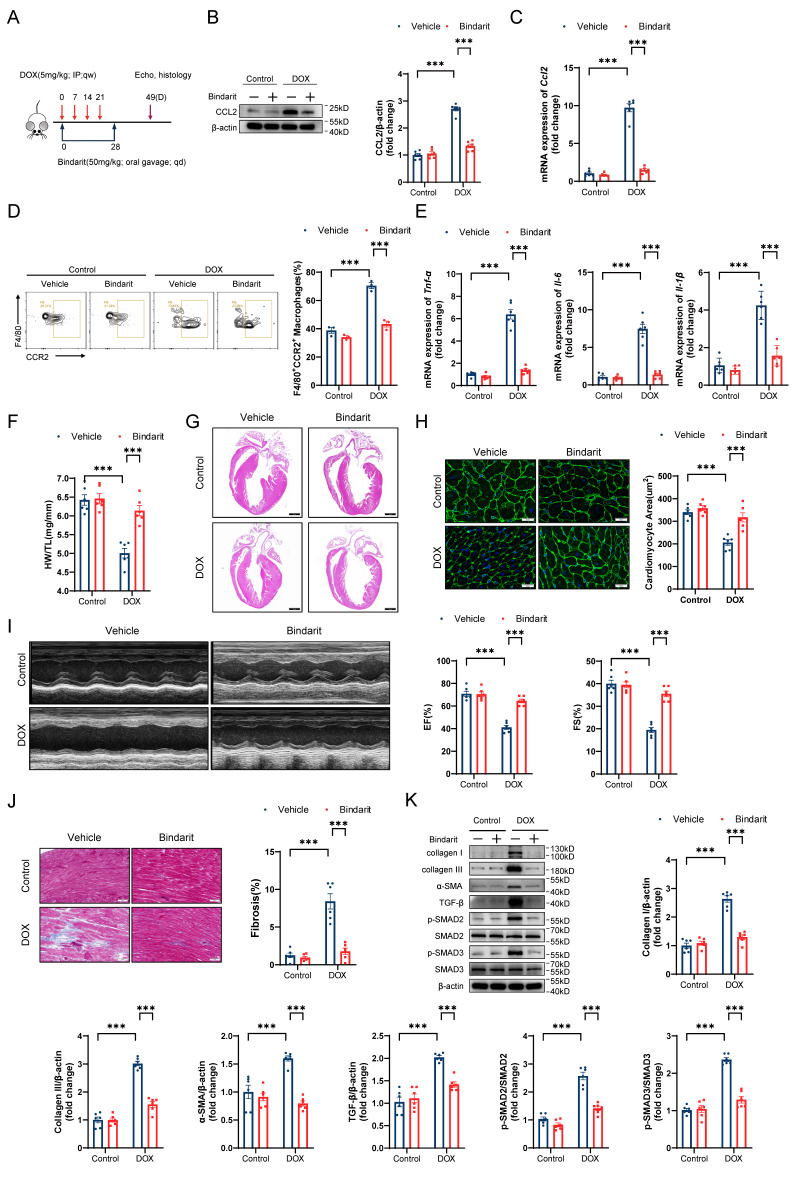
** The CCL2 inhibitor bindarit prevents DIC. A,** Bindarit intervention strategy. **B,** Representative immunoblotting and relative quantitative analysis of mouse heart CCL2 expression; n = 6 per group. **C,** RT‒qPCR analysis of *Ccl2* transcription levels in mouse hearts; n = 6 per group. **D,** Representative flow cytometry images and quantitative analysis of CD45+CD11b+F4/80+CCR2+ macrophages in the heart; n = 3 per group. **E,** RT‒qPCR analysis of the transcription levels of *Tnf-α*, *Il-6,* and *Il-1β* in mouse hearts; n = 6 per group. **F,** Ratio of HW to TL; n = 6 per group. **G,** Representative H&E staining of mouse hearts. Scale bar = 1000 μm. **H,** Representative WGA staining of mouse hearts and quantitative analysis of the cross-sectional area of cardiomyocytes. Scale bar = 20 μm; n = 6 per group. **I,** Representative echocardiography of mouse hearts and measurements of EF and FS; n = 6 per group. **J,** Representative Masson's trichrome staining and quantitative results of mouse hearts. Scale bar = 50 μm; n = 6 per group. **K,** Representative immunoblotting and relative quantitative analysis of collagen I, collagen III, α-SMA, TGF-β, p-SMAD2, SMAD2, p-SMAD3 and SMAD3 expression in mouse hearts; n = 6 per group. *** p < 0.001.

To elucidate the role of CCL2 in DIC, we treated DIC-induced mice with the CCL2 inhibitor bindarit (50 mg/kg) by oral gavage for 4 weeks (Figure 4A). As expected, bindarit markedly reduced the transcriptional and translational levels of cardiac CCL2 on day 14 after DOX administration (Figure 4B and 4C). Flow cytometry and RT‒qPCR revealed that bindarit inhibited DOX-induced CCR2+ macrophage infiltration and cardiac inflammation (Figure 4D and 4E). On day 49 after DOX administration, we evaluated pathological alterations in the heart. Bindarit efficiently attenuated the DOX-induced decreases in heart weights, heart sizes, and cardiomyocyte areas (Figure 4F through 4H). Moreover, compared with control (solvent-treated) DIC model mice, bindarit-treated DIC model mice exhibited greater left ventricular EF and FS (Figure 4I). Masson's trichrome staining and immunoblotting revealed that the DOX-induced upregulation of cardiac fibrosis and fibrosis-related proteins was ameliorated by bindarit (Figure 4J and 4K). These results suggest a cardioprotective role of CCL2 inhibition.

### CCR2+ macrophage depletion prevents DIC

**Figure 5:**
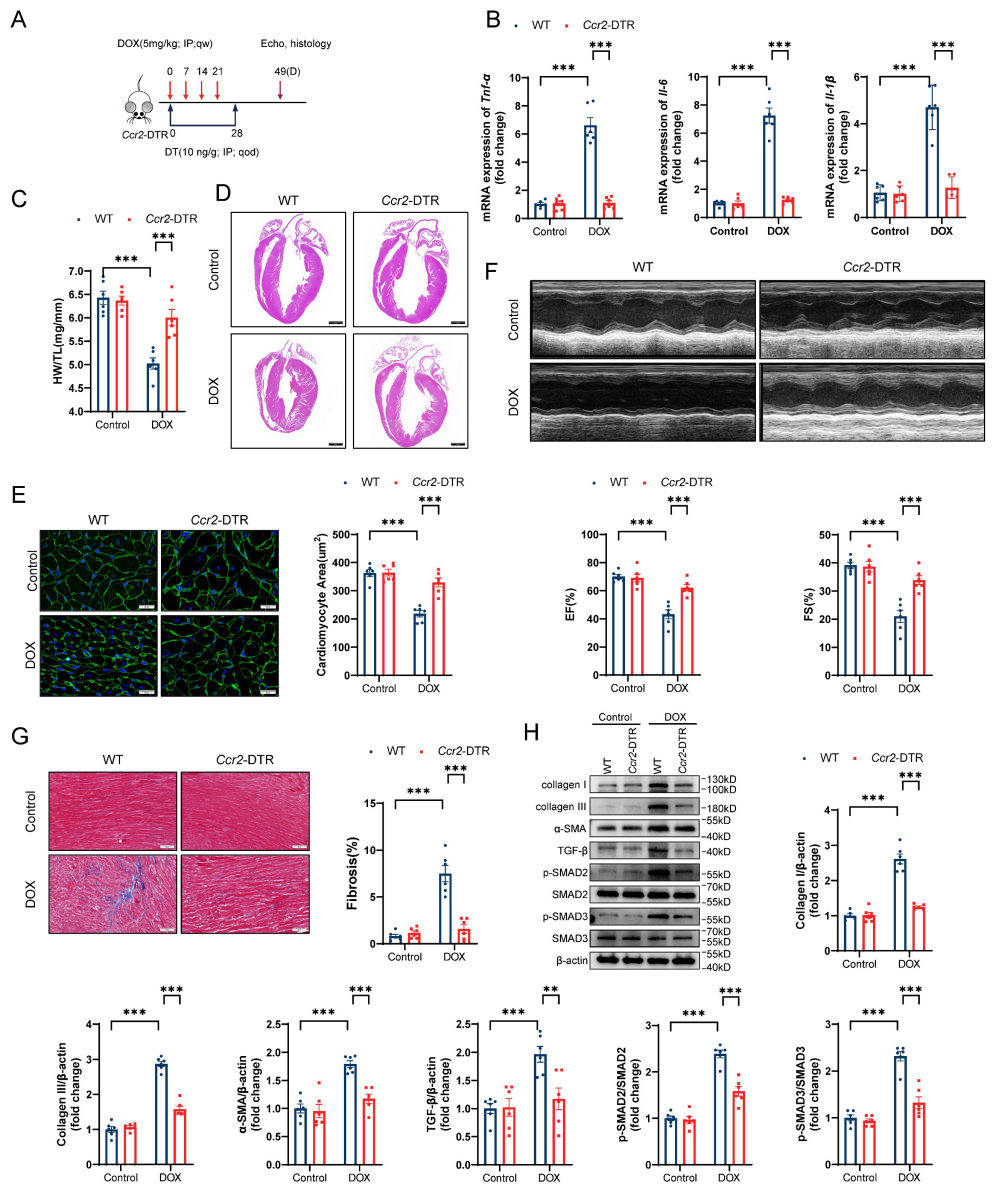
** CCR2+ macrophage depletion prevents DIC. A,** Schematic illustration of CCR2+ macrophage depletion by DT injection every two days for 4 consecutive weeks in a DIC model.** B,** RT‒qPCR analysis of the mRNA levels of *Tnf-α*, *Il-6,* and *Il-1β* in mouse hearts; n = 6 per group. **C,** HW/TL; n =6 per group. **D,** Representative H&E staining of hearts from WT and *Ccr2*-DTR mice upon DOX treatment. Scale bar = 1000 μm. **E,** Representative images of WGA staining and quantification of cardiomyocyte cross-sectional area in heart sections from WT and *Ccr2*-DTR mice upon DOX administration. Scale bar = 20 μm; n = 6 per group. **F,** Representative M-mode echocardiography of mouse heart and measurements of left ventricular EF and FS in WT and *Ccr2*-DTR mice upon DOX treatment; n = 6 per group. **G,** Representative images of Masson's trichrome staining and quantification of cardiac fibrosis in heart sections from WT and *Ccr2*-DTR mice receiving DOX. Scale bar = 50 μm; n = 6 per group. **H,** Representative immunoblot and summary data showing the expression levels of collagen I, collagen III, α-SMA, TGF-β, phospho-SMAD2, total-SMAD2, phospho-SMAD3, and total-SMAD3 in the hearts of WT and *Ccr2*-DTR mice upon DOX administration; n = 6 per group. **P < 0.01, ***P < 0.001.

To precisely delineate the influence of CCR2+ macrophages on DIC pathogenesis, we incorporated *Ccr2*-DTR (diphtheria toxin receptor) strain mice into our experimental design (Figure S4A and S4B). *Ccr2-*DTR mice have been previously established to exhibit depletion of peripheral blood CCR2+ monocytes and cardiac-resident CCR2+ macrophages after diphtheria toxin (DT) administration^48^. Following DT administration, there was an obvious decrease in the number of both circulating and cardiac-resident CCR2+ monocytes/macrophages during the first two days, as indicated by flow cytometry (Figure S4C and S4D). Four days after DT administration, CCR2+ monocytes/macrophages reappeared as circulating cells due to their rapid renewability (Figure S4C and S4D). On the basis of these findings, *Ccr2-*DTR mice received DT via intraperitoneal injection once every 2 days for 4 consecutive weeks in the DIC model (Figure 5A). CCR2+ macrophage depletion markedly inhibited cardiac inflammation after doxorubicin treatment (Figure 5B). Compared with WT mice, *Ccr2-*DTR mice showed a preserved heart weights, heart sizes, and cardiomyocyte cross-sectional areas (Figure 5C through 5E). Impaired cardiac systolic function, indicated by the reduction in EF and FS in DOX-treated mice, was recovered in *Ccr2-*DTR mice (Figure 5F). CCR2+ macrophage depletion markedly suppressed myocardial fibrosis, collagen deposition, and aberrant TGF-β/SMAD2/SMAD3 signaling activation in the hearts of DIC mice (Figure 5G and 5H). These data suggest that CCR2+ macrophage depletion played a cardioprotective role in DIC.

### Cardiomyocyte-specific *Ccl2* overexpression reverses the cardioprotective role of *Gsdme* knockout in DIC

As a rescue assay, we injected DIC-treated *Gsdme^CKO^* mice with cardiomyocyte-specific *Ccl2*-overexpressing AAV9 (Figure S5A). Forced expression of CCL2 in *Gsdme^CKO^* murine hearts was confirmed by immunoblotting and RT‒qPCR (Figure S5B and S5C). Flow cytometry and RT‒qPCR revealed that the inhibition of CCR2+ macrophage infiltration and the inflammatory response in DOX-treated *Gsdme^CKO^* murine hearts was abolished by *Ccl2* overexpression (Figure S5D and S5E). Compared with AAV9-Null-injected *Gsdme^CKO^* mice, AAV9-*Ccl2*-OE-injected *Gsdme^CKO^* mice showed reduced heart weights, heart sizes, and cardiomyocyte areas in DIC (Figure S5F through S5H). Moreover, the recovery of cardiac systolic function, as indicated by increased left ventricular EF and FS in *Gsdme^CKO^* mice, was inhibited by cardiomyocyte-specific *Ccl2* overexpression in DIC (Figure S5I). Masson's trichrome staining and immunoblotting revealed worsened cardiac fibrosis and upregulated fibrosis-related protein expression in AAV9-*Ccl2*-OE-injected *Gsdme^CKO^* mice compared with AAV9-Null-injected *Gsdme^CKO^* mice in DIC (Figure S5J and S5K). These results reveal the important role of the CCL2-CCR2 axis in the promotion of DIC by GSDME.

### *Gsdme* knockout protects against DOX-induced mitochondrial damage and mtDNA release

**Figure 6:**
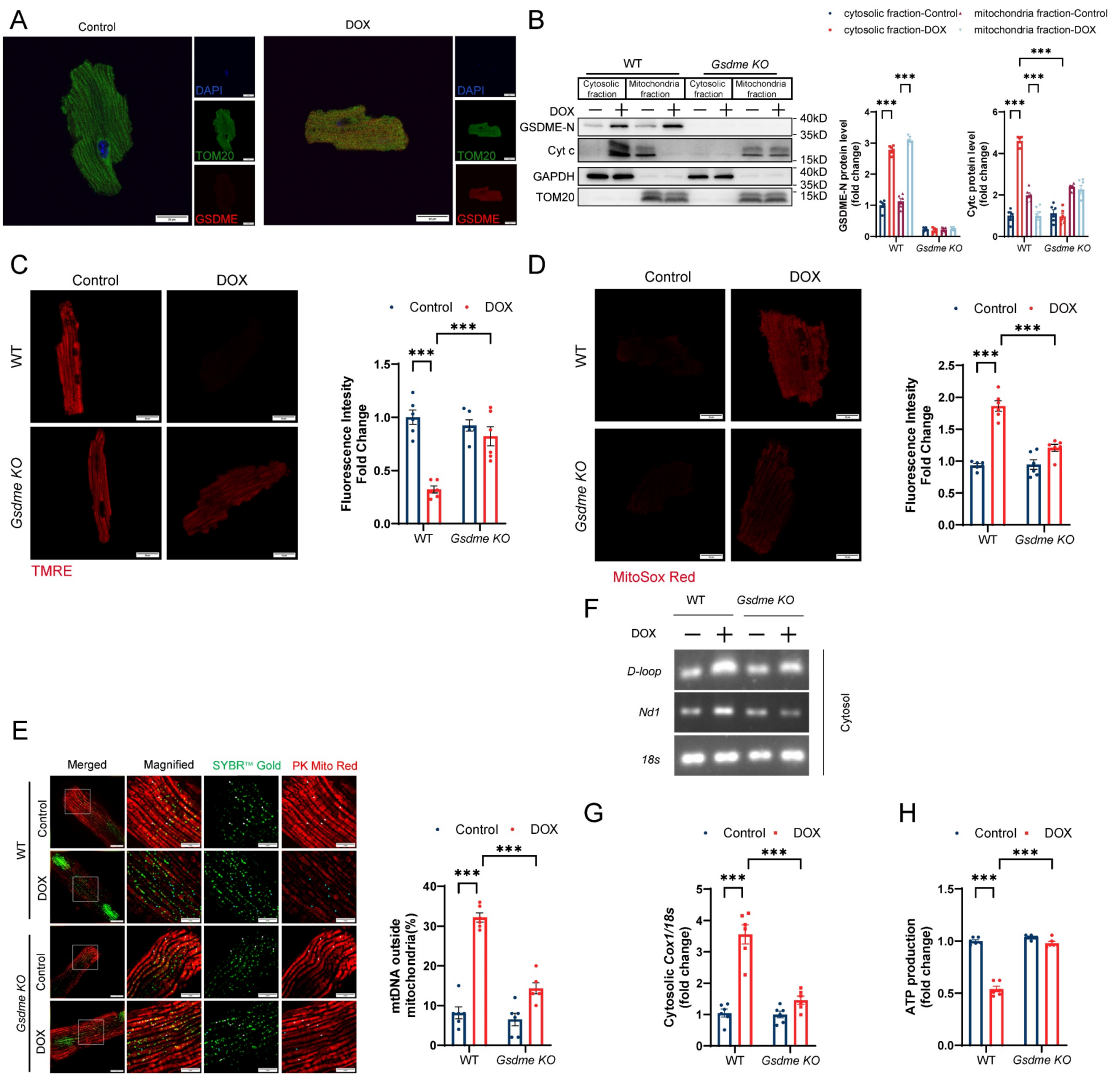
***Gsdme* knockout protects against DOX-induced mitochondrial damage and mtDNA release. A,** Representative immunofluorescence staining of GSDME and TOM20 in WT AMCMs 24 hours after DOX stimulation. Scale bar = 20 μm. **B,** Representative immunoblots and quantification of the intensity of cleaved N-terminal GSDME and Cyt c bands from cytosolic and mitochondrial fractions from WT and *Gsdme-KO* AMCMs treated with DOX for 24 hours; n = 6 per group. **C,** Representative fluorescence images of TMRE staining in WT and *Gsdme-KO* AMCMs 24 hours after DOX treatment. Scale bar = 20 μm; n = 6 per group. **D,** Representative fluorescence images of MitoSOX Red-stained WT and *Gsdme-KO* AMCMs 24 hours after DOX treatment. Scale bar = 20 μm; n = 6 per group. **E,** Representative images of extra-mitochondrial (PK Mito Red) mtDNA (SYBR^TM^ Gold) in WT and *Gsdme-KO* AMCMs stimulated with DOX for 24 hours. Scale bar = 10 μm. The white arrowheads represent mtDNA inside the mitochondria, whereas the blue arrowheads indicate mtDNA outside the mitochondria; n = 6 per group. **F and G,** Cytosolic mtDNA levels in DOX-treated WT and *Gsdme-KO* AMCMs detected by primers for *D-loop* or *Nd1* using PCR **F)** and by a primer for *Cox1* using qPCR **G)**. Nuclear DNA detected by a primer for *18s* was used for normalization; n = 6 per group. **H,** ATP production in cardiomyocytes measured using a firefly luciferase-based ATP assay kit; n = 6 per group. ***P < 0.001.

GSDME has been reported to bind the plasma membrane as well as the mitochondrial membrane^36^. Immunofluorescence staining confirmed that DOX stimulation resulted in the localization of GSDME to mitochondria (Figure 6A). Immunoblot analysis of fractionated lysates revealed that DOX upregulated the level of cleaved N-terminal GSDME in both the cytosolic and mitochondrial fractions of WT AMCMs but not in those of* Gsdme-KO* AMCMs (Figure 6B). Cleaved GSDME can insert into and form pores on the mitochondrial membrane.

Therefore, we examined whether GSDME enrichment induced mitochondrial damage. The mitochondrial membrane potential (MMP) plays an indispensable role in maintaining mitochondrial biological processes. We employed TMRE, a voltage-sensitive mitochondrial dye, to visualize the disruption of the MMP. We discovered that DOX triggered slight TMRE accumulation in WT AMCMs, indicating mitochondrial depolarization; this effect was notably mitigated in *Gsdme-KO* AMCMs (Figure 6C). Cytochrome c (Cyt c), which is located in the mitochondrial intermembrane/intercristal spaces, serves as a crucial indicator of outer mitochondrial membrane damage when it is released into the cytosol^49^. Compared with WT AMCMs, *Gsdme-*KO AMCMs displayed less Cyt c release after DOX treatment, suggesting that the outer membrane impairment was alleviated (Figure 6B). DOX also induced excessive production of mitochondrial reactive oxygen species (ROS), as reflected by enhanced signal of the mitochondria-specific superoxide fluorescent probe MitoSOX Red in WT AMCMs. However, *Gsdme* knockout inhibited ROS accumulation (Figure 6D). We next detected the DOX-induced mitochondrial DNA (mtDNA) release into the cytoplasm by multimodality structured illumination microscopy, PCR, and qPCR. Immunofluorescence staining revealed a significant increase in mtDNA outside the mitochondria in DOX-treated WT AMCMs, which was greatly attenuated by *Gsdme* knockout (Figure 6E). *Gsdme* knockout also mitigated the marked increase in the cytosolic mtDNA content (*D-loop, Nd1*) in AMCMs after DOX stimulation (Figure 6F). DOX stimulation increased the ratio of cytosolic mtDNA (*Cox1*) to nuclear DNA (*18S*) in WT AMCMs, but this increase was effectively reversed in *Gsdme-KO* AMCMs (Figure 6G). *Gsdme* knockout also improved mitochondrial function in doxorubicin-treated cardiomyocytes, as indicated by increased ATP production (Figure 6H). Collectively, these results reveal the critical role of GSDME in mitochondrial damage and mtDNA release by DOX.

### STING/NFκB mediates GSDME-induced regulation of CCL2 production

**Figure 7:**
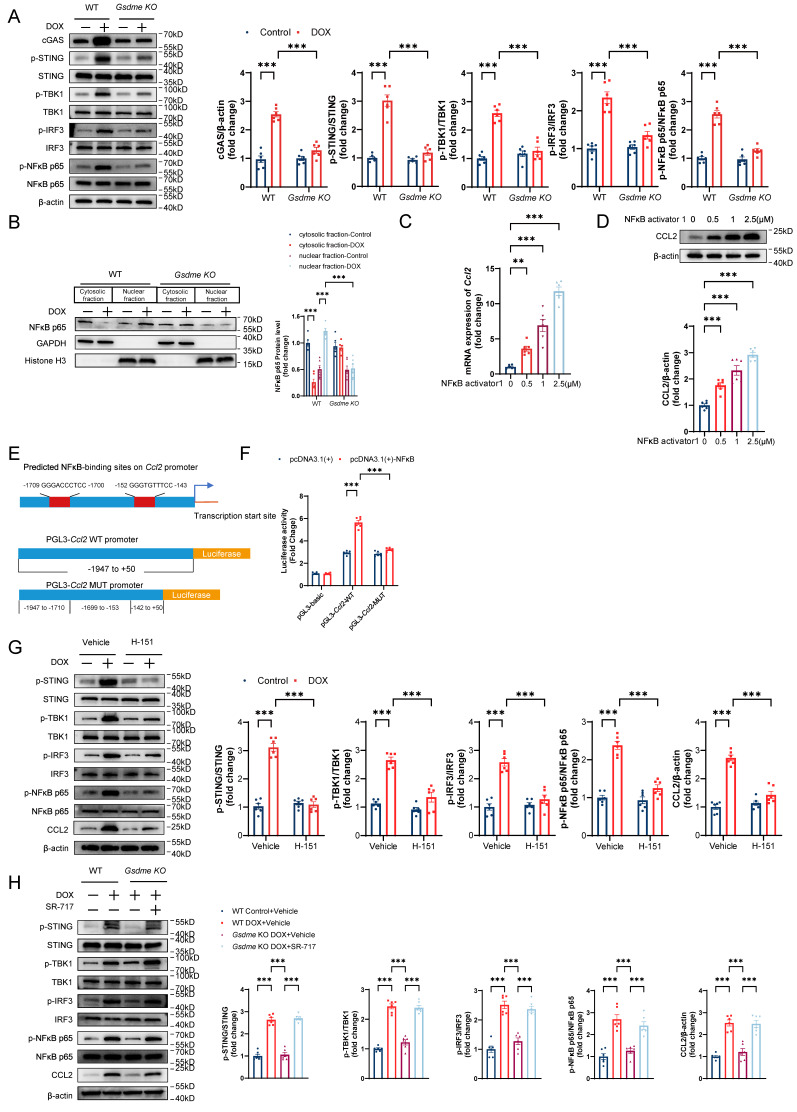
** STING/NFκB mediates regulation of CCL2 production by GSDME. A,** Representative western blot and quantitative analysis of STING/NFκB-related proteins in DOX-treated WT and *Gsdme-KO* AMCMs; n = 6 per group. **B,** Representative immunoblot images and quantification of NFκB p65 in cytosolic and nuclear fractions from DOX-treated WT and *Gsdme-KO* AMCMs; n = 6 per group. **C and D,** RT‒qPCR **C)** and immunoblotting **D)** analysis of CCL2 in NFκB activator 1-treated AMCMs; n = 6 per group **E,** NFκB-binding sites on the promoter region of *Ccl2* predicted using the JASPAR database and luciferase reporter plasmids with cloning of the WT or mutant *Ccl2* promoter sequence. **F,** Luciferase activity after the cotransfection of NFκB overexpression plasmids and WT or mutant *Ccl2* promoter luciferase reporter plasmids. Firefly luciferase activity was normalized to that of Renilla luciferase; n = 6 per group. **G and H,** Representative immunoblots and summary of STING/NFκB-related proteins and CCL2 in AMCMs treated with H-151 **G)** or SR-717 **H)**; n = 6 per group. **P < 0.01, ***P < 0.001.

Cytoplasmic mtDNA is recognized by the pattern recognition receptor cGAS, which activates the STING/NFκB pathway and promotes the production of multiple inflammatory factors and the recruitment of inflammatory cells^50^, ^51^. Therefore, we investigated whether the STING/NFκB pathway is involved in the GSDME-mediated regulation of the CCL2-CCR2 axis. *Gsdme* knockout inhibited the upregulation of STING/NFκB pathway proteins, including cGAS, phospho-STING, phospho-TBK, phospho-IRF3, and phospho-NFκB p65, in DOX-treated AMCMs (Figure 7A). Increased NFκB P65 nuclear translocation was also observed in WT AMCMs after DOX stimulation but was reversed in *Gsdme-KO* AMCMs (Figure 7B). After incubation with the NFκB agonist NFκB activator 1, AMCMs showed dose-dependent CCL2 upregulation at the mRNA and protein levels (Figure 7C and 7D). Dual-luciferase reporter assays revealed that CCL2 was directly transcriptionally regulated by NFκB (Figure 7E and 7F). H-151, a STING inhibitor, inhibited STING/NFκB activation as well as CCL2 production (Figure 7G), whereas the small-molecular STING agonist SR-717 reversed *Gsdme* knockout-induced inactivation of STING/NFκB and CCL2 loss in DOX-treated AMCMs (Figure 7H). These findings suggest that the STING/NFκB pathway mediated the regulation of CCL2 production by GSDME.

### The STING inhibitor H-151 prevents DIC

**Figure 8:**
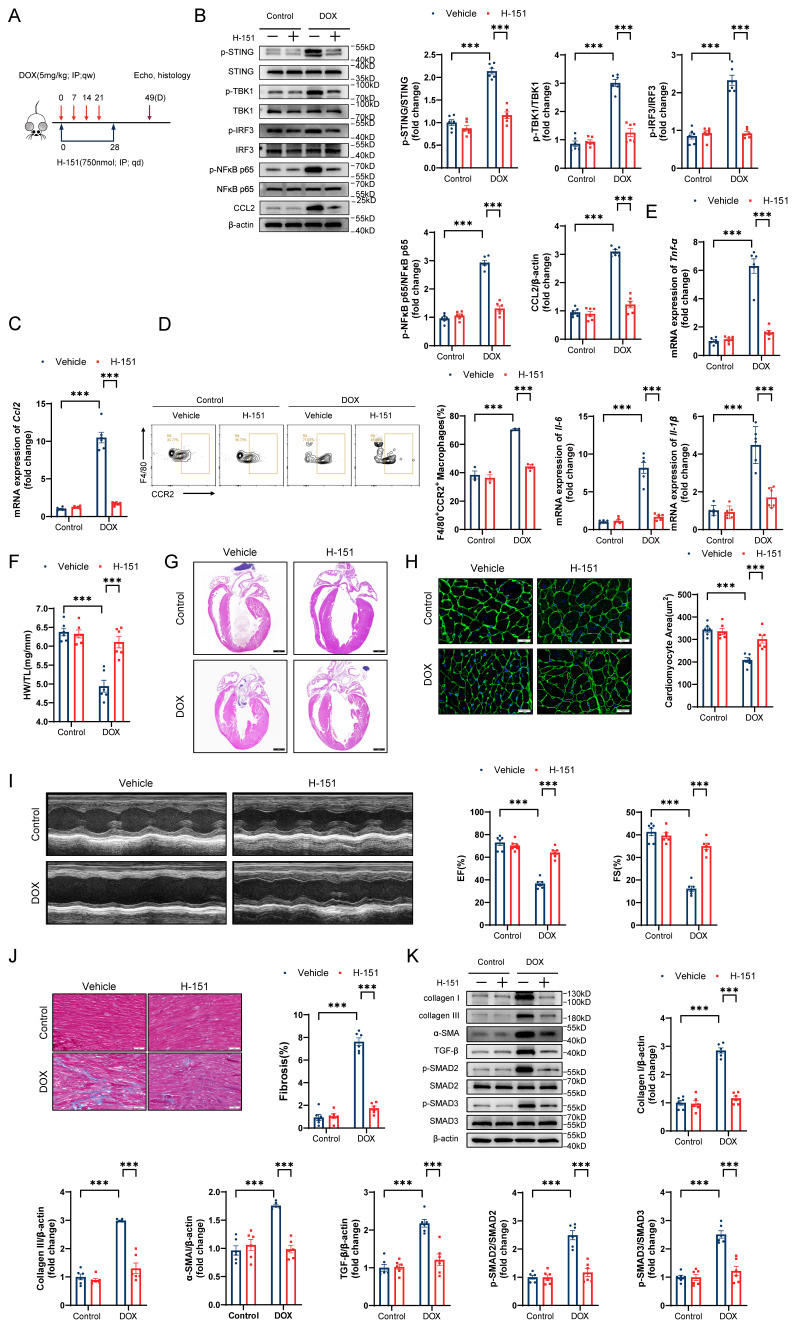
** The STING inhibitor H-151 prevents DIC. A,** Diagram of the H-151 intervention strategy. **B,** Representative western blots and statistical results of p-STING/STING, p-TBK1/TBK1, p-IRF3/IRF3, p-NFκB p65/NFκB p65 and CCL2 in the hearts of H-151-treated DIC mice; n = 6 per group. **C,** RT‒qPCR analysis of *Ccl2* transcriptional levels in the hearts of H-151-treated DIC mice; n = 6 per group. **D,** Representative flow cytometry images and quantitative analysis of CD45+CD11b+F4/80+CCR2+ macrophages in the hearts of H-151-treated DIC mice; n = 3 per group. **E,** RT‒qPCR analysis of the transcriptional levels of *Tnf-α*, *Il-6,* and *Il-1β* in the hearts of H-151-treated DIC mice; n = 6 per group. **F,** Ratio of HW to TL; n = 6 per group. **G,** Representative H&E staining of the hearts of H-151-treated DIC mice. Scale bar = 1000 μm. **H,** Representative WGA staining of mouse hearts and statistical results of the cardiomyocyte cross-sectional area. Scale bar = 20 μm; n = 6 per group. **I,** Representative M-mode images and echocardiographic measurements of the left ventricular EF and FS in H-151-treated DIC mice; n = 6 per group. **J,** Representative Masson's trichrome staining and quantitative results of H-151-treated hearts. Scale bar = 50 μm; n = 6 per group. **K,** Representative immunoblotting and quantitative histograms of collagen I, collagen III, α-SMA, TGF-β, p-SMAD2, SMAD2, p-SMAD3 and SMAD3 in H-151-treated hearts; n = 6 per group. ***P < 0.001.

To further explore the role of the STING/NFκB pathway in the GSDME-mediated promotion of DIC *in vivo*, we first observed that *Gsdme* knockout attenuated DOX-induced mitochondrial swelling, crest disintegration, fracture, and disappearance and STING/NFκB activation on day 14 after DOX injection (Figure S6A and S6B). We subsequently treated DIC model mice with the STING inhibitor H-151 via intraperitoneal injection for 4 weeks (Figure 8A). Immunoblotting and RT‒qPCR confirmed STING/NFκB inactivation and reduced CCL2 levels in H-151-treated DIC model mice (Figure 8B and 8C). Flow cytometry and RT‒qPCR revealed that H-151 weakened cardiac CCR2+ macrophage infiltration and inflammation (Figure 8D and 8E). On day 49 in the DIC model, compared with the solvent control, H-151 preserved heart weights, heart sizes, and cardiomyocyte cross-sectional areas (Figure 8F through 8H). H-151 also improved cardiac systolic function in DOX-treated mice (Figure 8I). Alleviation of heart fibrosis, collagen deposition, and TGF-β/SMAD2/SMAD3 activation was observed in H-151-treated DIC model mice (Figure 8J and 8K). These findings reveal that STING inhibition prevented DIC.

### The STING agonist SR-717 reverses the cardioprotective role of *Gsdme* knockout in DIC

We next clarified the impact of SR-717 on DIC model *Gsdme^CKO^* mice. *Gsdme^CKO^* mice received SR-717 via intraperitoneal injection daily at a dose of 10 mg/kg for 4 consecutive weeks (Figure S7A). On day 14 of DIC modeling, we found that the inactivation of the STING/NFκB pathway and inhibition of CCL2 expression induced by *Gsdme* knockout were diminished by SR-717 administration in DIC (Figure S7B and S7C). Similarly, SR-717 aggravated cardiac CCR2+ macrophage recruitment and inflammation in DIC model *Gsdme^CKO^* mice (Figure S7D and S7E). On day 49 after DOX administration, the preservation of heart weights, heart sizes, and cardiomyocyte cross-sectional areas in DIC *Gsdme^CKO^* mice was decreased by SR-717 (Figure S7F through S7H). Specifically, SR-717 administration aggravated cardiac insufficiency in DOX-treated *Gsdme^CKO^* mice (Figure S7I), and the attenuation of myocardial fibrosis, collagen deposition, and abnormal TGF-β/SMAD2/SMAD3 activation in DOX-treated *Gsdme^CKO^* mice was suppressed by SR-717 administration (Figure S7J and S7K). Taken together, these findings confirm that STING activation reversed the cardioprotective role of *Gsdme* knockout in DIC.

### Pharmacological blockade of GSDME alleviates DIC

**Figure 9:**
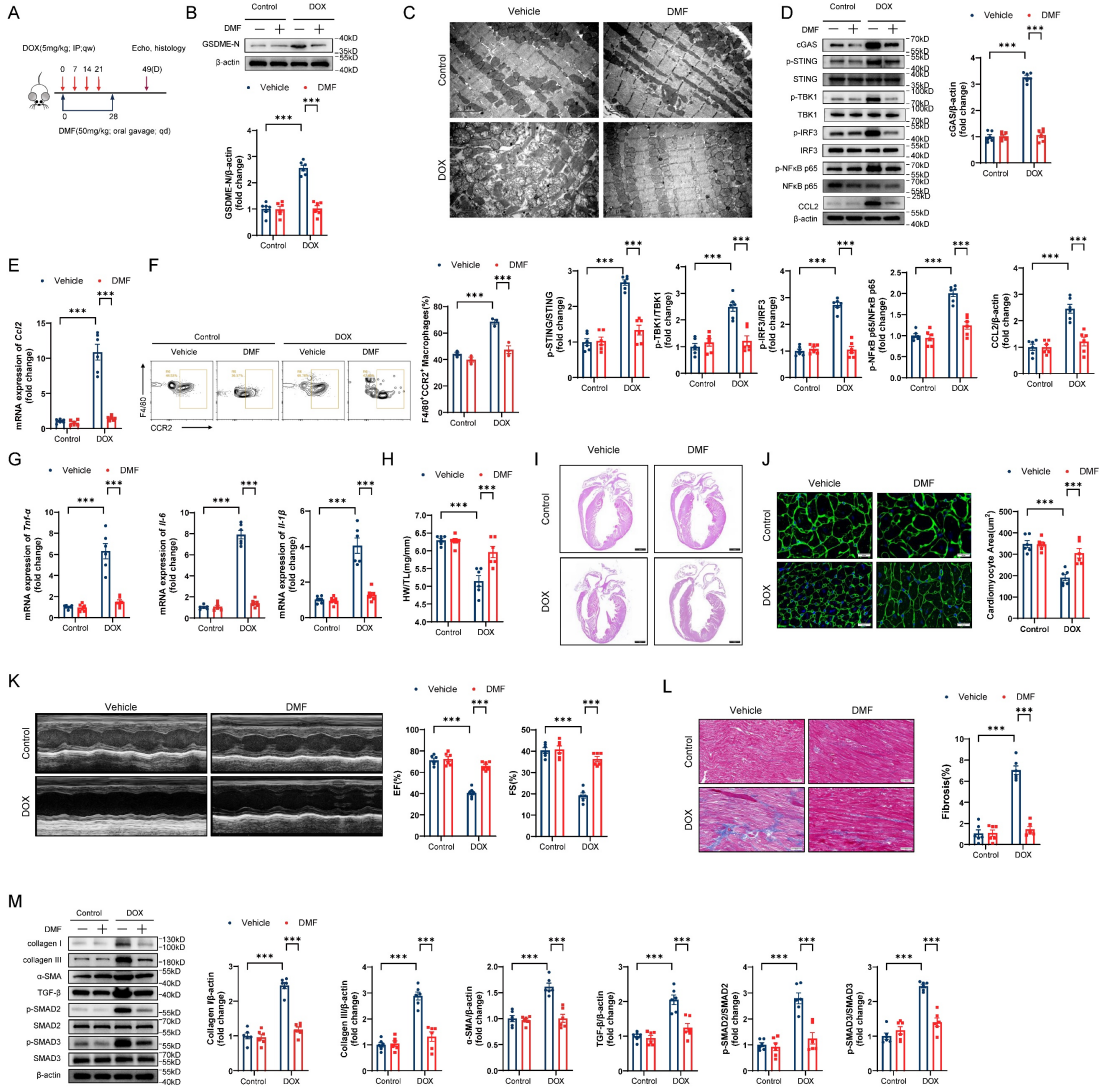
** Pharmacological blockade of GSDME alleviates DIC. A,** Diagram of the experimental design of DMF administration via oral gavage in a chronic DIC model. **B,** Representative western blots and statistical histograms of cleaved GSDME in the hearts of DMF-treated DIC mice; n = 6 per group. **C,** Representative transmission electron microscopy images of mouse cardiomyocytes. Scale bar = 2 μm. **D,** Representative western blots and gray-intensity measurements of p-STING/STING, p-TBK1/TBK1, p-IRF3/IRF3, p-NFκB p65/NFκB p65 and CCL2 in the hearts of DMF-treated DIC mice; n = 6 per group. **E,** RT‒qPCR detection of *Ccl2* mRNA levels in the hearts of DMF-treated DIC mice; n = 6 per group. **F,** Representative flow cytometric images and statistical histograms of CD45+CD11b+F4/80+CCR2+ macrophages in the hearts of DMF-treated DIC mice; n = 3 per group. **G,** RT-qPCR detection of the mRNA levels of *Tnf-α*, *Il-6,* and *Il-1β* in the hearts of DMF-treated DIC mice; n = 6 per group. **H,** Ratio of HW to TL; n = 6 per group. **I,** Representative H&E staining of hearts from DMF-treated DIC mice. Scale bar = 1000 μm. **J,** Representative WGA staining and quantitative analysis of the cardiomyocyte cross-sectional area in the hearts of DMF-treated DIC mice. Scale bar = 20 μm; n = 6 per group. **K,** Representative M-mode echocardiography and quantification of cardiac systolic functions, including left ventricular EF and FS, in DMF-treated DIC mice; n = 6 per group. **L,** Representative Masson's trichrome staining and quantification of myocardial fibrosis in the hearts of DMF-treated DIC mice. Scale bar = 50 μm; n = 6 per group. **M,** Representative immunoblot and quantitative analysis of collagen I, collagen III, α-SMA, TGF-β, phospho-SMAD2, total-SMAD2, phospho-SMAD3, and total-SMAD3 in the hearts of DMF-treated DIC mice; n = 6 per group. ***P < 0.001.

Given the potential for clinical translation of this approach, we tested the effect of pharmacological blockade of GSDME in a DIC model (Figure 9A). Administration of the GSDME inhibitor DMF successfully abrogated GSDME cleavage in DIC murine hearts (Figure 9B). On day 14 of DIC modeling, DMF not only attenuated mitochondrial damage but also inhibited STING/NFκB activation and CCL2 production (Figure 9C through 9E). Moreover, cardiac CCR2+ macrophage infiltration and inflammation decreased after DMF treatment (Figure 9F and 9G). On day 49 after DOX injection, the DOX-induced reduction in heart weights, heart sizes, and cardiomyocyte cross-sectional areas was mitigated in response to DMF (Figure 9H through 9J). Improved left ventricular EF and FS were observed in DMF-treated DIC model mice, indicating excellent recovery of cardiac function in DIC (Figure 9K). Notable reductions in the cardiac fibrosis area, collagen deposition, and TGF-β/SMAD2/SMAD3 activation were also observed in DMF-treated DIC model mice (Figure 9L and 9M). Collectively, these results indicate that the pharmacological blockade of GSDME alleviated DIC.

## Discussion

This study presents three novel findings. First, we confirmed that global or conditional *Gsdme* knockout in cardiomyocytes but not in myeloid cells attenuated pathological alterations in DIC. Second, we clarified that the CCL2-CCR2 axis mediated the ability of GSDME to promote DIC, while both CCL2 inhibition and CCR2+ macrophage depletion exerted a cardioprotective effect on DIC. Third, we confirmed that the STING/NFκB pathway was involved in the GSDME-mediated regulation of the CCL2-CCR2 axis.

The increased levels of full-length and cleaved GSDME in DOX-treated murine hearts observed in our study are consistent with findings from previous studies^42^-^44^. However, those previous studies were more focused on the upstream molecules that regulate pyroptosis in DIC, regarding GSDME simply as a marker of pyroptosis. *Gsdme^-/-^* mice have been studied in various cardiovascular disease contexts^40^, ^41^, ^52^, but not yet in DIC. In our study, *Gsdme*^-/-^ mice exhibited a cardioprotective phenotype in DIC, as demonstrated by the significant attenuation of cardiac remodeling, cardiac malfunction, and cardiac fibrosis. Intriguingly, conditional *Gsdme* knockout in cardiomyocytes, rather than myeloid cells, replicated the cardioprotective role in *Gsdme*^-/-^ mice, although some studies have indicated that GSDME is predominantly expressed in macrophages and involved in their biological functions^40^. Through an analysis of two public single-cell RNA sequence datasets from mouse heart tissues, Sun *et al.* found that GSDME was enriched in cardiomyocytes and fibroblasts, unlike GSDMD in immune cells, providing evidence to support cardiomyocytes as a major source of GSDME^41^. Our findings are also in accordance with the aforementioned notion that, unlike GSDMD, GSDME is abundantly expressed in normal tissue^36^ and suggest that cardiomyocytes rather than interstitial cells might be the main site through which GSDME functions in DIC. Another well-known gasdermin protein, GSDMD, has been proven to have a detrimental effect by impairing mitochondria and promoting cardiomyocyte apoptosis in DIC^35^. However, no significant upregulation of full-length GSDMD or cleavage N-terminal GSDMD in DIC heart tissue was observed in another study^42^. We speculate that this controversial result might be ascribed to the difference in dosage administration and harvest time point. However, determining the importance of GSDME and GSDMD in DIC is difficult because GSDME has the ability to induce different types of programmed cell death^36^, and the functions of GSDMD might involve cross-talk with those of GSDME. However, further investigation is needed to test this linkage. Notably, a gain-of-function mutation in GSDME, known as deafness autosomal dominant 5, is associated with nonsyndromic hearing impairment^53^. Therefore, this might be due to the difference in genetic predisposition, as some patients develop DIC, whereas others may be resistant to higher doses of DOX. Further studies on whether gain-of-function mutations affect cardiomyocyte fate are warranted. Moreover, further efforts should consider the genetic variations in patients when DOX is prescribed in clinical practice.

Cardiac inflammation is among the mechanistic hallmarks of DIC. We found that at the early stage of DIC, the levels of CCL2, TNF-α, IL-6, and IL-1β in the heart of mice were significantly elevated, which was similar to previously reported results^21^, ^23^-^25^, indicating that multiple inflammatory factors are involved in the progression of DIC. Ni *et al.* reported that IFN-γ metabolically reprogrammed cardiomyocytes by inhibiting AMPK signaling, leading to increased cardiomyocyte vulnerability^54^, whereas temporarily blocking IFN-γ improved DIC without affecting antitumor effects^55^. TNF-α may promote cardiomyocyte apoptosis via the TRAF3-TAK1-MAPK axis^56^, while *Il-6* mRNA levels are predictive and protective factors of DIC^57^. IL-1β is commonly regarded as a marker of pyroptosis. The CCL2-CCR2 axis is a major chemokine signaling pathway and is associated with the progression of multiple cardiovascular diseases, such as heart failure, atherosclerosis, coronary heart disease, hypertension, and myocardial disease^58^. GSDME regulates mononuclear macrophages and inflammation in various disease models^41^, ^59^, especially in myocarditis associated with aPD-1 treatment, as the increased proportion of cardiac CCR2+ macrophages was inhibited in *Gsdme^-/-^* mice^41^. Therefore, we hypothesized that, in addition to mediating pyroptosis, GSDME might modulate the CCL2-CCR2 axis in DIC. Encouragingly, we found that cardiomyocyte-conditional *Gsdme* knockout significantly reduced the DOX-induced increase in cardiac CCL2 levels and the proportion of CCR2+ macrophages. This provided a preliminary basis for our hypothesis. Further investigation revealed that the CCL2 inhibitor bindarit significantly blocked DOX-induced cardiac CCL2 production and CCR2+ macrophage infiltration, weakened cardiac inflammation, and alleviated DIC, whereas CCR2+ macrophage depletion had a similar cardioprotective effect on DIC. CCR2+ macrophages might play a key role in GSDME-mediated DIC through their proinflammatory potential. Gambardella *et al.* reported that DOX induces macrophages to release catecholamines, leading to mitochondrial apoptosis of cardiomyocytes through β-AR stimulation^60^. In a left ventricular pressure overload model, CCL2-mediated macrophage recruitment induces myocardial fibrosis through a TGF-β-mediated process. Neutralizing CCL2 inhibits macrophage infiltration, TGF-β induction, and fibroblast proliferation while alleviating diastolic dysfunction and myocardial fibrosis^61^. Other possible biological processes include macrophage metabolic reprogramming^21^, cytokine release and chemotactic signal generation, monocyte and neutrophil recruitment, cardiomyocyte signal transduction^62^, ^63^, and cardiac fibroblast activation^61^; however, further evidence is needed to confirm the involvement of these processes. Notably, Wang *et al.* reported that exercise plays an important anti-inflammatory role in DIC through the upregulation of FC-γ receptor IIB expression in B cells^46^. Another study revealed that TP53-mediated treatment-related clonal hematopoiesis promoted DIC by enhancing neutrophil-mediated cytotoxic responses^64^. High levels of neutrophil traps in plasma are also associated with DIC^7^. Activation of invariant natural killer T cells by alpha-galactosylceramide has been reported to improve DIC in mice^65^. Therefore, complex relationships may exist among immune cells and between immune and nonimmune cells in DIC, but these relationships need to be verified by further studies.

As a pyroptosis-related protein, GSDME can permeate not only the plasma membrane but also the mitochondrial membrane, similar to GSDMD^41^, ^66^. To investigate the role of GSDME in cardiomyocytes *in vitro*, we first examined the subcellular localization of GSDME following DOX treatment. We observed a translocation of GSDME to the mitochondria, which caused mitochondrial damage, including MMP impairment, Cyt c release, excessive mitochondrial ROS production, and mtDNA leakage in AMCMs. Notably, the STING/NFκB pathway, which senses intracellular DNA to promote the innate immune response, plays a critical role in these processes^67^. Mounting evidence has shown that the STING pathway is activated in multiple cardiovascular diseases, including cardiac hypertrophy, ICI-induced myocarditis, myocardial infarction, and heart failure^41^, ^68^-^70^. Recent research has emphasized the activation of the STING pathway in endothelial cells and cardiomyocytes in DIC^71^-^74^. Generally, after binding to dsDNA, cGAS catalyzes the synthesis of cGAMP, which binds to STING to activate TANK-binding kinase 1 (TBK1); this results in the phosphorylation of interferon regulatory factor 3 (IRF3) and NFκB, which in turn activates the transcription of type I interferons and other proinflammatory cytokines, such as CCL2, IL6, and TNF-α^75^-^77^. *In vivo* experiments demonstrated that the STING inhibitor H-151 effectively suppressed STING/NFκB pathway activation and CCL2 production in cardiomyocytes and mouse hearts, thereby reducing CCR2+ macrophage infiltration and cardiac inflammation and improving cardiac pathological changes in DIC. In our rescue experiment, the STING agonist SR-717 activated the STING/NFκB pathway and the CCL2-CCR2 axis and increased the inflammatory response in the hearts of *Gsdme^CKO^* mice with DIC, reversing the protective effect of cardiomyocyte-conditional *Gsdme* knockout in DIC. These findings confirmed that GSDME regulated the CCL2-CCR2 axis through the STING/NFκB pathway in DIC. Finally, to test the clinical translation potential of this approach, we examined the therapeutic effect of the GSDME inhibitor DMF on DIC. Notably, the administration of 50 mg/kg DMF once a day to mice can significantly alleviate DOX-induced pathological changes, providing a strong basis for future clinical drug development.

It should be acknowledged that the current study has several limitations. For ethical reasons, we were unable to examine the cardiac GSDME expression pattern in patients receiving DOX regimens. DT injection in *Ccr2-*DTR mice eliminated not only monocytes/macrophages but also dendritic cells^78^; therefore, the contribution of dendritic cells to the orchestration of the immune context in DOX-induced hearts may have been overlooked, although dendritic cells represent only a small proportion of cardiac immune cells. In addition, cardiac tissue-resident CCR2- macrophages reduce adverse cardiac remodeling in DIC^21^. Therefore, further investigations are needed to determine whether there is communication between CCR2- and CCR2+ macrophages in DIC. Further investigations are needed to investigate the tumor-bearing model of DIC and the cardioprotective strategies against DIC while not affecting the antitumor efficacy of doxorubicin.

While GSDME is a tumor suppressor, it is also a cause of doxorubicin-induced cardiotoxicity. This initially seems paradoxical; however, the underlying mechanism is the same, as GSDME mediates cytotoxic effects in both cases. The key difference lies in the desired outcomes—we aim to induce tumor cell death while preserving normal tissue survival. Similarly, we aim to activate the STING pathway in tumor cells to trigger antitumor immunity while avoiding harmful autoimmune responses in normal tissues. This ultimately hinges on achieving tissue specificity in therapy, that is, precisely targeting tumor tissues while minimizing the impact on healthy tissues. With new technological advances, the precise delivery of antitumor drugs via oncolytic viruses or nanoparticles appears to be able to not only treat cancer but also minimize side effects on normal tissues^79^, ^80^. However, further clinical studies are still needed to validate their efficacy. Given that systemic chemotherapy drugs remain widely used in clinical practice, developing protective agents for multiple organs also seems to be a promising approach.

In conclusion, we found that GSDME induces mitochondrial damage, causing mtDNA leakage and STING/NFκB pathway activation, thereby activating the CCL2-CCR2 axis, aggravating cardiac inflammation, and ultimately leading to DIC. These findings emphasize the possibility of targeting GSDME for the prevention and treatment of DIC.

## Supplementary Material

Supplementary methods and figures.
