# Supplementary material for: Cardiomyocyte GSDME Drives Doxorubicin-Induced Cardiotoxicity by Targeting the CCL2-CCR2 Axis
Source: Int J Biol Sci. 2025 Sep 29;21(14):6113–31. doi: 10.7150/ijbs.115489 (PMC12594575; doi:10.7150/ijbs.115489)
Supplement: Supplementary file 1 — Supplementary methods and figures. [file ijbsv21p6113s1.pdf]

Supplementary materials

Cardiomyocyte GSDME Drives Doxorubicin-Induced Cardiotoxicity by Targeting the CCL2–CCR2 Axis

Methods

Mice

All animal experiments complied with the ARRIVE guidelines and with the Guidelines for the Care and Use of Laboratory Animals published by the US National Institutes of Health (NIH Publication No. 85–23, revised 1996).

Mouse strains, including WT, *Gsdme*$^{-/-}$, *Gsdme*$^{f/f}$, *Myl2*-Cre, *Lyz2*-Cre, and *Ccr2*-DTR strains, were purchased from Shanghai Model Organisms Center, Inc. (Shanghai, China). Exon 4 of the *Gsdme* gene was the targeted region for knockout or being flanked by loxP recombination sites. *Gsdme*$^{f/f}$ mice were crossed with *Myl2*-Cre mice or *Lyz2*-Cre mice to generate *Gsdme*^{CKO} (cardiomyocyte-specific *Gsdme* knockout) or *Gsdme*^{MKO} (macrophage-specific *Gsdme* knockout) mice and littermate-negative mice (*Gsdme*^{Con}). Mice were housed and maintained on a 12-hour light/dark cycle in a specific pathogen-free temperature- and humidity-controlled facility with free access to standard chow and tap water *ad libitum*. All mutations described above were on the C57BL/6J background and were genotyped according to established protocols. The primers used for genotyping were as follows:

Gsdme$^{-/-}$: (forward) 5’-3’ TTGGGGCGGGAAAGGTC and (reverse) 5’-3’ AAGCAGGGCAGTTACAGGAG

Gsdme$^{f/f}$: (forward) 5’-3’ GTTCTGCATGCTGGGCTAGA and (reverse) 5’-3’ CAATAGCAAGGTCCTGGGGG

Myl2-Cre: (forward) 5’-3’ AATGGCAGGGAAGAGAGCAC and (reverse) 5’-3’

GTTGTTCAGCTTGCACCAGG

Lyz2-Cre: (Mutant) 5'-3' CCCAGAAATGCCAGATTACG and (Common) 5'-3' CTTGGGCTGCCAGAATTTCTC and (Wild type) 5'-3' TTACAGTCGGCCAGGCTGAC

Ccr2-DTR:

(P1) 5'-3' CCATGCAGGTGACAGAGACT and (P2) 5'-3' TGCTGCTACTTCATAGCTGTAAT

(P3) 5'-3' TCTCTGCAAACAGTGCCCAG and (P4) 5'-3' TTTGGCGAGAGGGGAAAGAC

To establish a chronic doxorubicin-induced cardiotoxicity (DIC) animal model, eight-week-old mice were administered DOX (#HY-15142A, MedChemExpress, NJ, USA) at a weekly dose of 5 mg/kg via intraperitoneal injection for 4 consecutive weeks for a cumulative dose of 20 mg/kg. The hearts were harvested at the indicated times (days 0, 3, 10, 14, 28, 42, and 49).

To explore the role of CCL2 inhibition in DIC, eight-week-old mice were given 50 mg/kg of the CCL2 inhibitor bindarit (#S3032; Selleck Chemicals, TX, USA) by oral gavage every day for 4 weeks.

To test the efficiency of CCR2+ monocyte/macrophage depletion, eight-week-old *Ccr2*-DTR mice received a single dose of 10 ng/g diphtheria toxin (DT; #D0564; Sigma–Aldrich, MO, USA) via intraperitoneal injection, and peripheral circulating CCR2+ monocytes and cardiac-resident CCR2+ macrophages were analyzed at the indicated times (days 0, 2, and 4). To explore the role of CCR2+ macrophage depletion

in DIC, DT was administered once every 2 days for 4 weeks via intraperitoneal injection in *Ccr2*-DTR mice in the chronic DIC model.

Cardiomyocyte-specific *Ccl2* overexpression was achieved via tail vein injection with an adeno-associated virus serotype 9 (AAV9)-based delivery vector carrying the cTnT promoter (Genomeditech, Shanghai, China). Briefly, AAV9-cTnT-*Ccl2* (5×10^{11} VG/mouse) was injected into five-week-old mice via the tail vein three weeks before the establishment of the chronic DIC model.

To decipher the role of STING inhibition, STING activation or DMF inhibition in DIC, 750 nmol H-151 (#S6652; Selleck Chemicals, TX, USA) by intraperitoneal injection or 10 mg/kg SR-717 (#HY-131454; MedChemExpress, USA) by intraperitoneal injection or 50 mg/kg DMF (#242926; Sigma–Aldrich, MO, USA) by oral gavage was administered to DIC mice daily for 4 weeks.

Histology

All mice were euthanized under deep anesthesia at the end of the experiment, and the hearts were obtained. The hearts were then fixed in 4% neutral paraformaldehyde (#G1101-500ML; Servicebio, Wuhan, China) overnight at 4°C. After being embedded in paraffin, the hearts were sectioned at 5 μm. Hematoxylin and eosin (H&E) staining, Masson's trichrome staining, and wheat germ agglutinin (WGA) staining were performed on the sections in accordance with general protocols. The stained sections were imaged using a DM2500 & DM2500 LED optical microscope (Leica, Wetzlar, Germany), and the images were analyzed with ImageJ 1.54f software (National

Institutes of Health, USA).

Echocardiography

Mice were anesthetized with 1.5% isoflurane through nose inhalation and monitored for respiratory rate and toe-pinch reflex in the supine position on a heated pad, and transthoracic echocardiography was performed using a Vevo 2100 high-resolution animal ultrasound imaging system (VisualSonics Inc., Toronto, Canada). 2D and M-mode echocardiographic images were acquired in the long- and short-axis views. Cardiac systolic function was evaluated by measuring left ventricular EF and FS with supporting software (Vevo 2100 v3.1.1; VisualSonics Inc., Canada).

Immunofluorescence

For immunofluorescence staining of the heart, the paraffin sections were subjected to a series of steps, including dewaxing, hydration, antigen retrieval, permeabilization, and blocking. Then, the sections were incubated with the following primary antibodies overnight at 4°C: anti-GSDME (#ARG42603, Arigo Biolaboratories, Hsinchu City, China), anti-cTnT (#564766, BD Biosciences, CA, USA), and anti-F4/80 (#565409, BD Biosciences, USA). Then, the sections were incubated with the corresponding secondary antibodies, including Alexa Fluor 594-conjugated donkey anti-rabbit IgG (#A-21207, Invitrogen, CA, USA), Alexa Fluor 488-conjugated goat anti-mouse IgG (#A-11001, Invitrogen, USA), and Alexa Fluor 488-conjugated donkey anti-rat IgG (#A-21208, Invitrogen, USA), for 1 hour at room temperature. The sections were finally

counterstained with 4',6-diamidino-2-phenylindole (DAPI, #P0131, Beyotime, Shanghai, China). The stained sections were imaged using a DM2500 & DM2500 LED optical microscope, and the images were analyzed with ImageJ 1.54f software.

Adult mouse cardiomyocytes (AMCMs) were cultured and stimulated as required on glass coverslips. After being washed with PBS, the cells were fixed using 4% neutral paraformaldehyde for 10 minutes at room temperature. After permeabilization and blocking, the cells were incubated with anti-GSDME antibody (#13075-1-AP; Proteintech, IL, USA) overnight at 4°C, followed by incubation with secondary Alexa Fluor 594-conjugated donkey anti-rabbit IgG antibody (#A-21207; Invitrogen, USA). Anti-TOM20 antibody (#CL488-11802; Proteintech, USA) was used to label the mitochondria. The nuclei were counterstained with DAPI. The cells were imaged under a laser confocal microscope (TCS SP5, Leica, Germany).

Cytokine array

The cytokine array was performed using a commercial ABplex Mouse Cytokine 15-Plex Assay Kit (#RK05203; ABclonal, Wuhan, China). Heart samples were homogenized in PBS containing PMSF. The homogenates were centrifuged at 5000×g for 10 minutes at 4°C, and the collected supernatants were appropriately diluted and used in the assay according to the manufacturer’s instructions.

Multiplexed flow cytometry

Mouse hearts were dissected and minced into small pieces in RPMI 1640

(#C11875500BT; Gibco, NY, USA) supplemented with a cocktail of collagenase II (2 mg/mL; #LS004176; Worthington, NJ, USA), collagenase XI (0.3 mg/mL; #C7657; Sigma–Aldrich, USA), DNase I (0.05 mg/mL; #DN25; Sigma–Aldrich, USA), and hyaluronidase (0.3 mg/mL; #H3506; Sigma–Aldrich, USA) and digested for 30 minutes at 37°C under mild shaking upside down. The cell suspensions were then passed through a 70-μm strainer (#abs7008; Absin, Shanghai, China) to remove undigested tissue. Red blood cell lysis buffer (#555899; BD Biosciences, USA) was used to lyse erythrocytes. Single-cell suspensions were preincubated with Fixable Viability Stain 510 (#564406; BD Biosciences, USA) to exclude dead cells and with anti-CD16/CD32 antibody (#553141; BD Biosciences, USA) to block Fc receptors. The cells were subsequently stained with a mixture of fluorescent antibodies against CD45 (#557659; BD Biosciences, USA), CD11b (#557960; BD Biosciences, USA), F4/80 (#566787; BD Biosciences, USA), and CCR2 (#150608; BioLegend, CA, USA) for 30 minutes at 4°C in the dark. Cell suspensions were detected using a NovoCyte flow cytometer (Agilent Technologies, CA, USA) and analyzed with NovoExpress software 1.6.0 (Agilent Technologies, USA).

For circulating immune cells in blood, pellets derived from blood samples by centrifugation at 500×g for 10 minutes were treated with red blood cell lysis buffer (#555899; BD Biosciences, USA), after which preincubation with fixable viability stain 510 (#564406; BD Biosciences, USA) and anti-CD16/CD32 antibody (#553141; BD Biosciences, USA) was performed. Resuspended single cells were stained with antibodies against CD45 (#557659; BD Biosciences, USA), CD11b (#557960; BD

Biosciences, USA), and CCR2 (#150605; BioLegend, USA) for 30 minutes in the dark at 4°C. Cell samples were acquired and analyzed as described above.

Adult mouse cardiomyocyte (AMCM) isolation and treatment

AMCMs were isolated using a well-established Langendorff-free method, as previously described[1]. Briefly, the descending aorta and inferior vena cava of fully anesthetized mice were transected, after which 7 mL of EDTA buffer was injected through the base of the right ventricle to clear as much blood as possible. The dissected heart with the ascending aorta clamped with Reynolds hemostatic forceps was immersed in EDTA buffer in a 60-mm dish, followed by sequential injection of EDTA buffer, perfusion buffer, collagen buffer (containing collagenase II (0.5 mg/mL; #LS004176; Worthington, USA), collagenase IV (0.5 mg/mL; #LS004188; Worthington, USA), and protease XIV (0.05 mg/mL; #P5147; Worthington, USA)) into the left ventricle. Once digestion was complete, the heart was dissociated into 1-mm pieces with forceps. The cell suspensions were then passed through a 100-μm cell strainer and allowed to settle by gravity for 20 minutes. For immediate harvest, 3 rounds of sequential gravity settling in perfusion buffer were performed, after which the myocyte-containing pellets and nonmyocytes from the combined supernatant fraction after every round of gravity settling were collected. For culture, three calcium reintroduction buffers with increasing proportions of Ca^{2+} instead of perfusion buffer were used in 3 rounds of sequential gravity settling. The settled myocytes were then resuspended in plating media and plated on culture dished precoated with laminin (5 μg/mL, #23017-15, Thermo Fisher

Scientific, MA, USA) in an incubator for 1 hour. Myocytes were then washed and incubated in culture media for further experiments.

For doxorubicin stimulation, AMCMs were treated with 1 μM doxorubicin immediately after removal of the plating media and harvested at the indicated times.

For caspase-3 inactivation, AMCMs were pretreated with 20 μM z-VAD (#HY-16658B; MedChemExpress, USA) for 30 minutes and then treated with a combination of 20 μM zVAD and doxorubicin for the indicated durations.

To inhibit or activate STING, AMCMs were pretreated with 0.5 μM H-151 or 3.6 μM SR-717 for 2 hours before doxorubicin stimulation.

To promote NFκB activity, AMCMs were pretreated with NFκB activator 1 (#HY-134476; MedChemExpress, USA) at the indicated concentration for 6 hours before doxorubicin stimulation.

Cell fractionation

The mitochondria and cytosol were isolated using a commercial kit (#C3601; Beyotime, China) according to the manufacturer's instructions. In short, the collected cells were incubated with isolation reagents on ice for 15 minutes and homogenized 10–30 times, followed by centrifugation at 1000×g for 10 minutes at 4°C. The transferred supernatants were centrifuged again at 1100×g for 15 minutes at 4°C to separate the mitochondrial fraction (in the pellet) and the cytosolic fraction (in the supernatant). To further purify the cytosol, the transferred supernatants containing the cytosol were centrifuged at 12000×g for 10 minutes at 4°C, after which the supernatants were

collected.

The isolation of nuclei and cytosol was performed utilizing another commercial cell fractionation kit (#ab109719; Abcam, MA, USA) according to the manufacturer's protocols.

Mitochondrial membrane potential (MMP), mitochondrial ROS and ATP detection

Tetramethylrhodamine, ethyl ester (TMRE, #C2001S, Beyotime, China) reagent was used to evaluate the MMP. Briefly, diluted TMRE (1:1000 dilution in TMRE assay buffer) was added to cultured AMCMs for 15 minutes at 37˚C, after which the cells were rinsed with prewarmed culture media 3 times. The MMP was evaluated by laser confocal microscopy at an excitation wavelength of 550 nm/emission wavelength of 575 nm.

MitoSOX Red (#S0061S; Beyotime, China) was used to measure the level of reactive oxygen species in the mitochondria. In brief, stimulated AMCMs were incubated with MitoSOX Red at a concentration of 5 μM (1:1000 dilution in PBS) for 15 minutes at 37˚C. Then, cells were washed twice with PBS, and images were acquired under the confocal microscope mentioned above at an excitation wavelength of 510 nm/emission wavelength of 580 nm.

Cellular ATP production was measured by a firefly luciferase-based ATP assay kit (#S0026; Beyotime, China) based on a previously described fluorescence technique (Genmed Scientifics, Inc.)[2].

Cytosolic dsDNA immunofluorescence, extraction and quantification

For cytosolic DNA detection, stimulated AMCMs were incubated with SYBR™ Gold (1:10000 dilution in prewarmed culture media, #S11494; Invitrogen, USA) and PK Mito Red (#PKMR-1; Genvivotech, Nanjing, China) at a concentration of 250 nM (1:1000 dilution in prewarmed culture media) for 15 minutes at 37°C. Images were captured with a multimodality structured illumination microscope (Multi-SIM, NanoInsights, Beijing, China) and analyzed with ImageJ 1.54f software.

Cytosolic DNA was extracted from the aforementioned cytosolic fraction using commercial DNeasy Blood and Tissue Kits for DNA Isolation (#69504; QIAGEN, Duesseldorf, Germany) according to the manufacturer's instructions. The level of cytosolic mtDNA was determined by PCR and qPCR. The primers for the mtDNA sequences encoding *D-loop*, *Nd1*, and *Cox1* and for the nuclear DNA sequence encoding *18s* ribosomal RNA were as follows:

D-loop: (forward) 5'→3' AATCTACCATCCTCCGTGAAACC and (reverse) 5'→3' TCAGTTTAGCTACCCCCAAGTTTAA

Nd1: (forward) 5'→3' AAACTATGTTCTCCGCCCCAA and (reverse) 5'→3' TGGAGTCAGTGCATTTTGGC

Cox1: (forward) 5'→3' GCCCCAGATATAGCATTCCC and (reverse) 5'→3' GTTCATCCTGTTCCTGCTCC

18s: (forward) 5'→3' TAGAGGGACAAGTGGCGTTC and (reverse) 5'→3' CGCTGAGCCAGTCAGTGT

Immunoblot

Murine heart tissue or AMCMs were lysed in ice-cold RIPA lysis buffer (#89900; Thermo Fisher Scientific, USA) supplemented with a protease inhibitor cocktail (#P1005; Beyotime, China). After centrifugation at 12000×g for 15 minutes at 4°C, the protein lysates were collected, and the protein concentrations were quantified by a BCA protein assay kit (#23225; Thermo Fisher Scientific, USA). The proteins were subsequently separated by SDS–PAGE and transferred to polyvinylidene fluoride (PVDF) membranes. Membranes were blocked with Protein Free Rapid Blocking Buffer (#PS108P, Epizyme Biotech, Shanghai, China) and incubated with primary antibodies against GSDME (#ab215191, Abcam, USA), cleaved N-terminal GSDME (#ab222407, Abcam, USA), caspase-3(#D3R6Y, Cell Signaling Technology, USA), collagen I (#ab260043, Abcam, USA), collagen III (#DY1299, Abways, Shanghai, China), a-SMA (#14395-1-AP, Proteintech, USA), TGF-β (#21898-1-AP, Proteintech, USA), phospho-SMAD2 (#AY0742, Abways, China), SMAD2 (#D43B4, Cell Signaling Technology, MA, USA), phospho-SMAD3 (#CY5140, Abways, China), SMAD3 (#CY5013, Abways, China), β-actin (# HRP-66009, Proteintech, USA), CCL2 (#BA1843-2, Boster, USA), Cyt c (#66264-1-Ig, Proteintech, USA), GAPDH (#AP0063, Bioworld Technology, Nanjing, China), TOM20 (#ab186735, Abcam, USA), cGAS (#A31676, Boster, USA), phospho-STING (#D8F4W, Cell Signaling Technology, USA), STING (#D2P2F, Cell Signaling Technology, USA), phospho-TBK1 (#D52C2, Cell Signaling Technology, USA), TBK1 (#CY5145, Abways, China), phospho-IRF3 (#29528-1-AP, Proteintech, USA), IRF3 (#CY5779, Abways, China),

phospho-NFκB p65 (#BY0127, Abways, China), NFκB p65 (#CY5034, Abways, China), and histone H3 (#17168-1-AP, Proteintech, USA) overnight at 4°C. After being washed, the membranes were incubated with secondary anti-mouse or anti-rabbit IgG antibodies conjugated with HRP for 1 hour at room temperature. Images were obtained with a ChemiDoc Imaging System (ChemiDoc MP, Bio-Rad, CA, USA) and analyzed with ImageJ 1.54f software.

Real-time reverse transcription–qPCR (RT–qPCR)

Total RNA from cardiomyocytes was extracted using RNA isolation reagent (#R401-01; Vazyme, Nanjing, China) according to the manufacturer's instructions. The purity and concentration were determined with a NanoDrop 2000 spectrophotometer (Thermo Fisher Scientific, USA). cDNA synthesis was performed using cDNA Synthesis SuperMix for qPCR (#11141ES60; Yeasen, Shanghai, China), and RT–qPCR was conducted with SYBR qPCR Master Mix (#Q711-02; Vazyme, China) on a CFX96 detection system (Bio-Rad, USA) using specific primers. *Actb* (#B662302; Sangon Biotech, Shanghai, China) was used as a reference gene. The relative mRNA levels were quantified using the $2^{-\Delta\Delta Ct}$ method. The sequences of primers used were as follows:

Ccl2: (forward) 5'→3' ACTCACCTGCTGCTACTCATTCAC and (reverse) 5'→3' TCTTTGGGACACCTGCTGCTG

Il-6: (forward) 5'→3' TTCTTGGGACTGATGCTGGTGAC and (reverse) 5'→3' CTGTTGGGAGTGGTATCCTCTGTG

Tnf-α: (forward) 5'→3' CCACCACGCTCTTCTGTCTACTG and (reverse) 5'→3'

TGGTTTGTGAGTGTGAGGGTCTG

Il-1β: (forward) 5'→3' TCGCAGCAGCACATCAACAAG and (reverse) 5'→3' TCCACGGGAAAGACACAGGTAG

Dual-luciferase reporter assay

HEK-293T cells (purchased from the National Collection of Authenticated Cell Cultures) were cultured to 70% confluence in 24-well plates and transfected with PGL3-*Ccl2*-mutant or WT promoter luciferase reporter plasmids (250 ng), NFκb overexpression or control plasmids (250 ng), and pRL-TK plasmids (25 ng) with a LIPOFECTAMINE™ 3000 transfection kit (#L3000015; Thermo Fisher Scientific, USA) according to the manufacturer's instructions. Plasmids were constructed by Genomeditech (Shanghai, China). After 48 hours, luciferase activity was detected using a dual-luciferase reporter assay system (#E1910; Promega, WI, USA) according to the manufacturer's instructions. Firefly luciferase activity was normalized to that of Renilla luciferase.

Transmission electron microscopy (TEM)

Isolated hearts were fixed in 2.5% glutaraldehyde at 4°C for 24 hours, followed by postfixation in 1% osmium tetroxide for 2 hours. After dehydration with an alcohol gradient, the samples were permeated and embedded in epoxy resin. The samples were sectioned at 60–80 μm and counterstained with 2% saturated uranyl acetate solution and 2.6% lead citrate solution. Images were captured with a transmission electron

microscope (FEI TECNAI Spirit, Thermo Fisher Scientific, MA, USA).

Statistical analysis

Statistical analyses were carried out in GraphPad Prism 10.2.2 software (GraphPad Prism Software Inc., CA, USA). All data are presented as the mean±SEM. The normality of data distributions was determined by the Shapiro–Wilk test, while the homogeneity of variance was determined by the F test. When normality and homogeneity of variance were satisfied, data analyses were conducted using two-tailed unpaired Student's *t* test for two independent groups or one-way or two-way ANOVA with post hoc Tukey's test for multiple groups or multiple variable comparisons, respectively; $p < 0.05$ was considered to indicate statistical significance.

Supplementary Figures

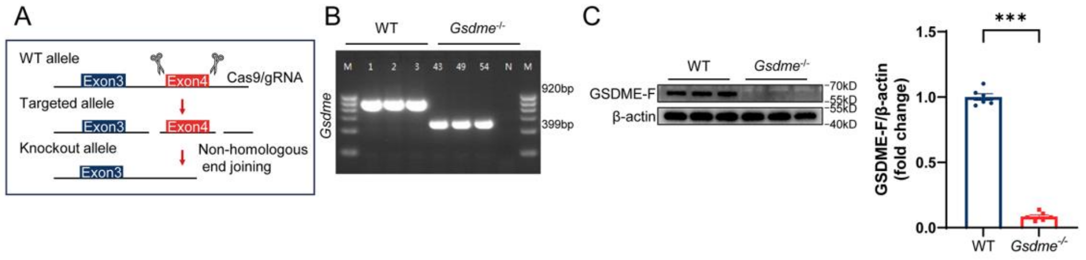

Figure S1. Generation of a global *Gsdme* knockout (*Gsdme*$^{-/-}$) mouse strain

A, Schematic of the generation of *Gsdme*$^{-/-}$ mice. **B,** Representative agarose image for genotyping *Gsdme*$^{-/-}$mice. **C,** Immunoblot images and quantification of GSDME in the hearts of WT and *Gsdme*$^{-/-}$ mice. ***P < 0.001.

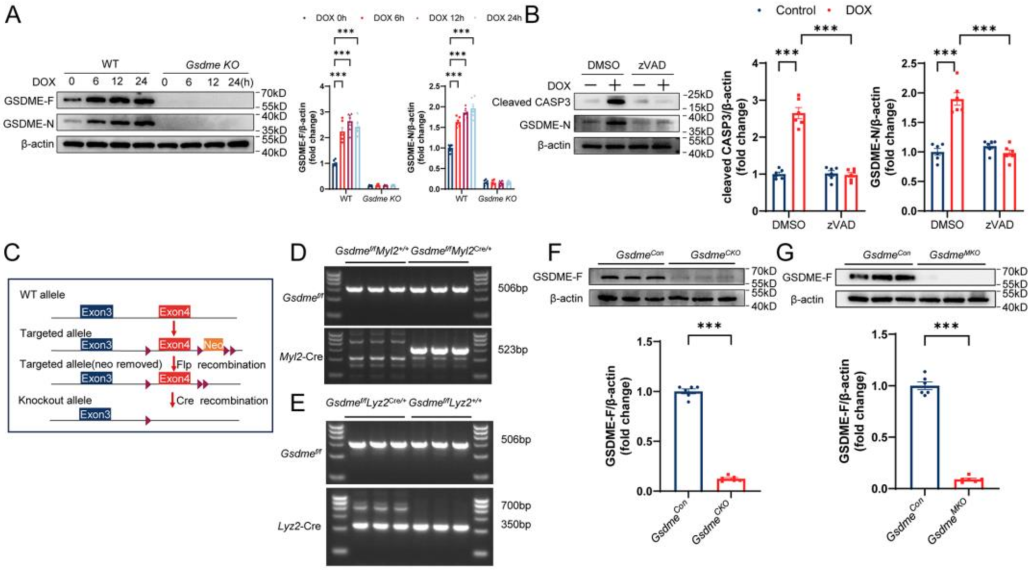

Figure S2. Generation of conditional *Gsdme* knockout in cardiomyocytes and myeloid cells

A, Representative immunoblotting and relative quantitative analysis of total GSDME and cleaved GSDME in WT and *Gsdme*-KO cardiomyocytes treated with DOX; n = 6 per group. **B,** Immunoblot images and quantification of cleaved caspase-3 and cleaved GSDME in doxorubicin-treated cardiomyocytes with or without zVAD; n = 6 per group. **C,** Schematic of the construction of *Gsdme*$^{f/f}$ mice. **D and E,** Representative agarose images for genotyping *Gsdme*^{CKO} **D)** and *Gsdme*^{MKO} **E)** mice. **F and G,** Protein level of GSDME in the hearts of *Gsdme*^{Con} and *Gsdme*^{CKO} mice **F)** and in bone marrow-derived macrophages from *Gsdme*^{Con} and *Gsdme*^{MKO} mice **G)**. ***P < 0.001.

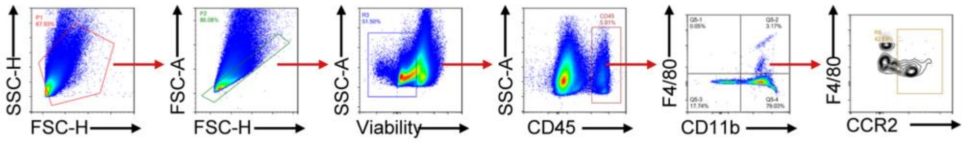

Figure S3. Gate strategy for generating mouse heart CD45+CD11b+F4/80+CCR2+ macrophages.

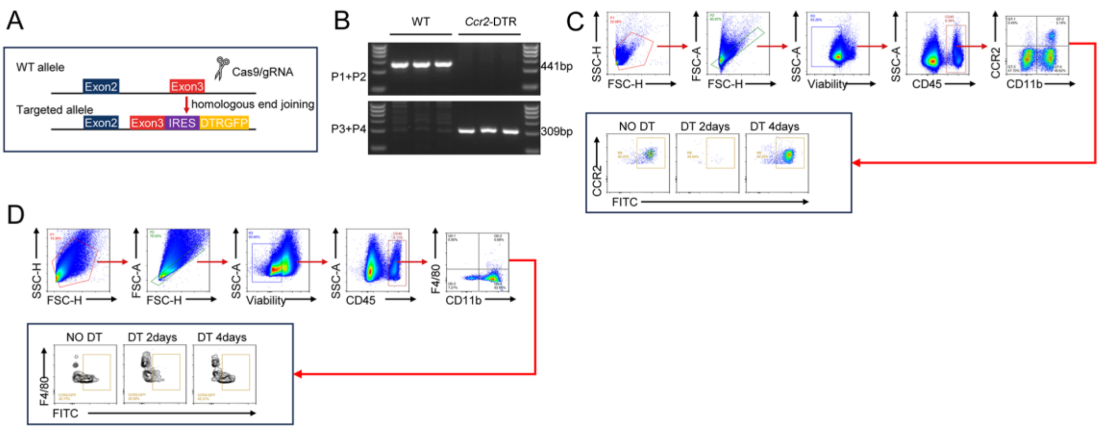

Figure S4. Generation of *Ccr2*-DTR mice and validation of CCR2+ macrophage depletion

A, Diagram of *Ccr2*-DTR mouse construction**. B,** Representative agarose images of genotyping for *Ccr2*-DTR mice. **C and D,** Dynamics of CCR2+ monocytes/macrophages in the blood **C)** and heart tissues **D)** of *Ccr2*-DTR mice after a single dose of diphtheria toxin.

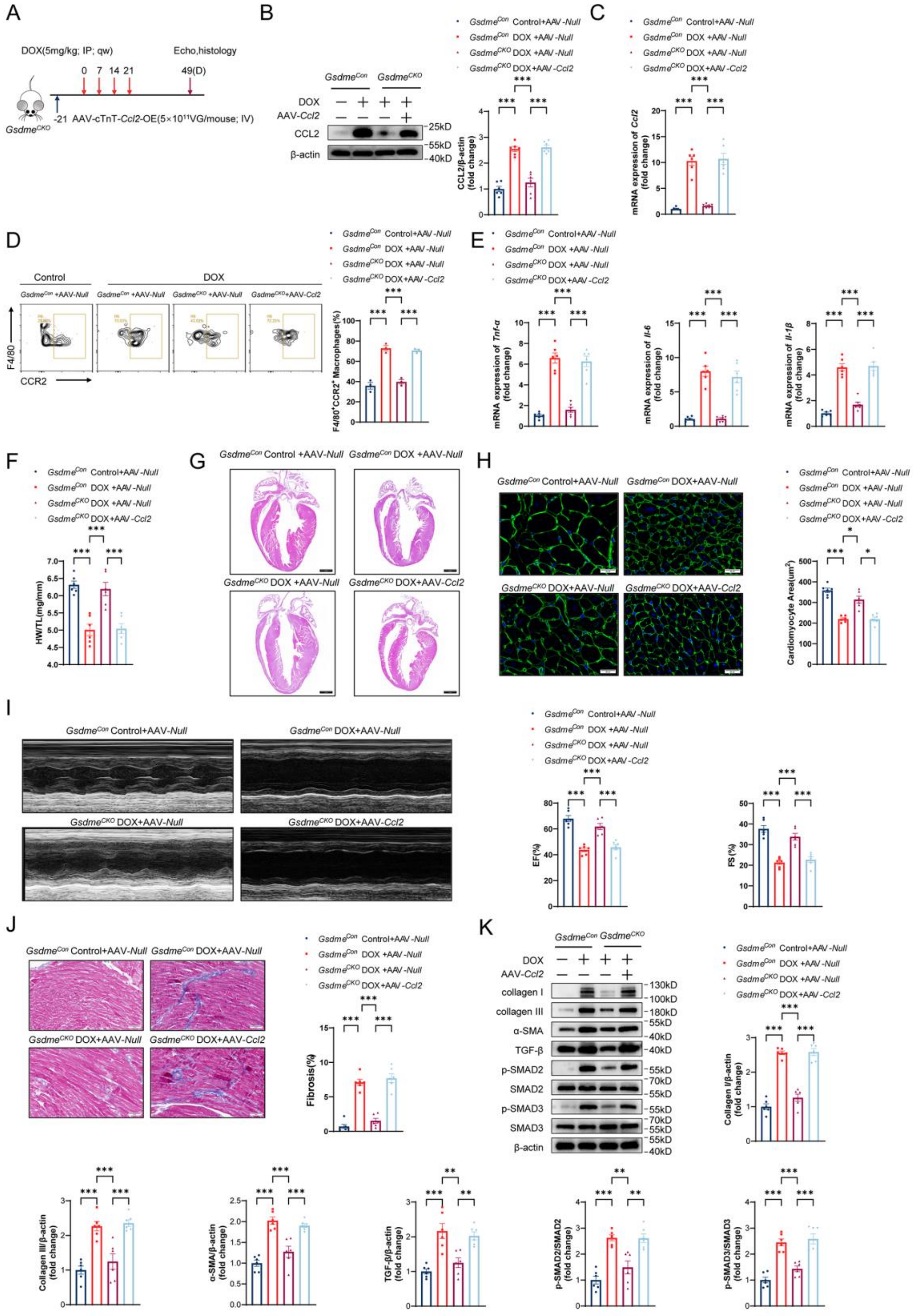
A
DOX(5mg/kg; IP; qw)
Echo,histology
0 7 14 21
49(D)
-21 AAV-cTnT-Ccl2-OE(5×10¹¹VG/mouse; IV)
Gsdme^CKO
B
Gsdme^Con
Gsdme^CKO
DOX
AAV-Ccl2
CCL2
β-actin
25kD
55kD
40kD
CCL2/β-actin (fold change)
Gsdme^Con Control+AAV-Null
Gsdme^Con DOX +AAV-Null
Gsdme^CKO DOX +AAV-Null
Gsdme^CKO DOX+AAV-Ccl2
C
mRNA expression of Ccl2 (fold change)
D
Control
DOX
Gsdme^Con +AAV-Null
Gsdme^CKO +AAV-Null
Gsdme^CKO+AAV-Ccl2
F4/80
CCR2
F4/80⁺CCR2⁺ Macrophages(%)
E
mRNA expression of Tnf-α (fold change)
mRNA expression of Il-6 (fold change)
mRNA expression of Il-1β (fold change)
F
HW/TL(mg/mm)
G
Gsdme^Con Control +AAV-Null
Gsdme^Con DOX +AAV-Null
Gsdme^CKO DOX +AAV-Null
Gsdme^CKO DOX+AAV-Ccl2
H
Gsdme^Con Control+AAV-Null
Gsdme^Con DOX+AAV-Null
Gsdme^CKO DOX+AAV-Null
Gsdme^CKO DOX+AAV-Ccl2
Cardiomyocyte Area(um²)
I
Gsdme^Con Control+AAV-Null
Gsdme^Con DOX+AAV-Null
Gsdme^CKO DOX+AAV-Null
Gsdme^CKO DOX+AAV-Ccl2
EF(%)
FS (%)
J
Gsdme^Con Control+AAV-Null
Gsdme^Con DOX+AAV-Null
Gsdme^CKO DOX+AAV-Null
Gsdme^CKO DOX+AAV-Ccl2
Fibrosis(%)
K
Gsdme^Con
Gsdme^CKO
DOX
AAV-Ccl2
collagen I
collagen III
α-SMA
TGF-β
p-SMAD2
SMAD2
p-SMAD3
SMAD3
β-actin
130kD
100kD
180kD
55kD
40kD
70kD
Collagen I/β-actin (fold change)
Collagen III/β-actin (fold change)
α-SMA/β-actin (fold change)
TGF-β/β-actin (fold change)
p-SMAD2/SMAD2 (fold change)
p-SMAD3/SMAD3 (fold change)

Figure S5. Cardiomyocyte-specific *Ccl2* overexpression abrogates the cardioprotective role of *Gsdme* knockout in DIC

A, Strategy for cardiomyocyte-specific overexpression of *Ccl2* in *Gsdme*^{CKO} mice. **B,** Representative immunoblotting and relative quantitative analysis of mouse heart CCL2 expression; n = 6 per group. **C,** RT–qPCR analysis of *Ccl2* mRNA levels in mouse hearts; n = 6 per group. **D,** Representative flow cytometry images and quantitative analysis of CD45+CD11b+F4/80+CCR2+ macrophages in the heart; n = 3 per group. **E,** RT–qPCR analysis of the transcription levels of *Tnf-α*, *Il-6* and *Il-1β* in mouse hearts; n = 6 per group. **F,** Ratio of HW to TL; n = 6 per group. **G,** Representative H&E staining of heart sections from DOX-treated *Gsdme*^{CKO} mice upon *Ccl2* overexpression. Scale bar = 1000 μm. **H,** Representative WGA staining and quantitative analysis of the cardiomyocyte cross-sectional area of DOX-treated *Gsdme*^{CKO} murine hearts upon *Ccl2* overexpression. Scale bar = 20 μm; n = 6 per group. **I,** Representative echocardiography and quantification of left ventricular EF and FS in DOX-treated *Gsdme*^{CKO} mice upon *Ccl2* overexpression; n = 6 per group. **J,** Representative Masson's trichrome staining and measurement of the myocardial fibrotic area in hearts from DOX-treated *Gsdme*^{CKO} mice upon *Ccl2* overexpression. Scale bar = 50 μm; n = 6 per group. **K,** western blot images and quantitative analysis of collagen I, collagen III, α-SMA, TGF-β, phospho-SMAD2, total-SMAD2, phospho-SMAD3, and total-SMAD3 in the hearts of DOX-treated *Gsdme*^{CKO} mice with *Ccl2* overexpression; n = 6 per group. $^{*}P < 0.05$, $^{**}P < 0.01$, $^{***}P < 0.001$.

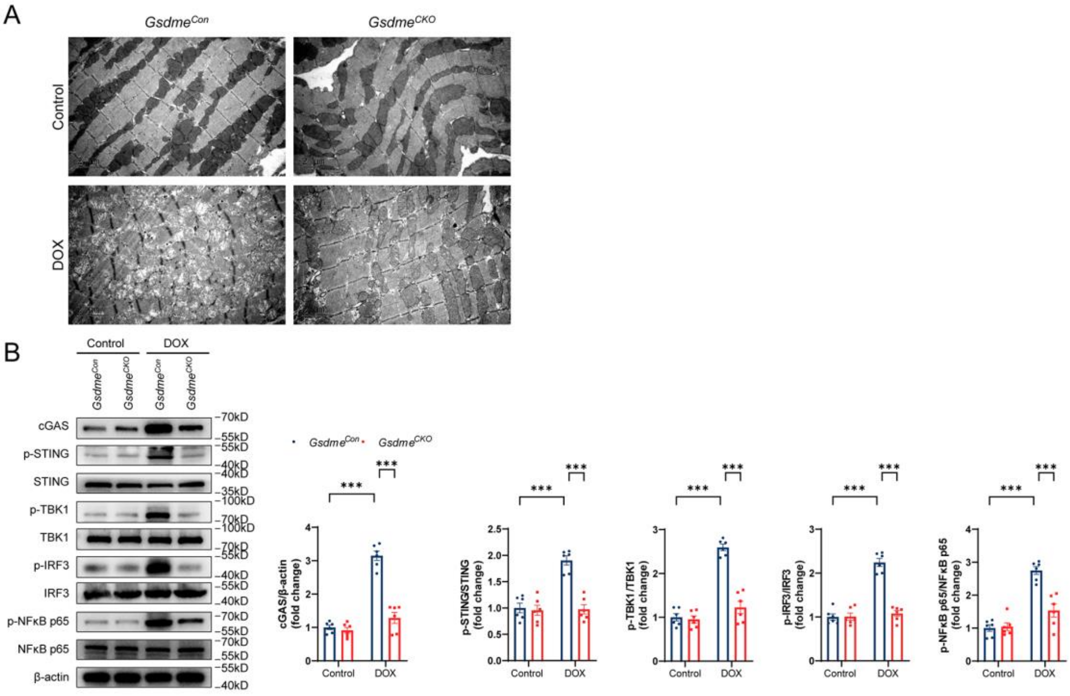

Figure S6. Gsdme knockout attenuates DOX-induced mitochondrial damage and STING/NFκB activation

A, Representative transmission electron microscopy images of cardiomyocyte mitochondria. Scale bar = 2 μm. **B,** Representative western blots and relative quantitative statistics of p-STING/STING, p-TBK1/TBK1, p-IRF3/IRF3, and p-NFκB p65/NFκB p65 in mouse hearts; n = 6 per group. *** $p < 0.001$.

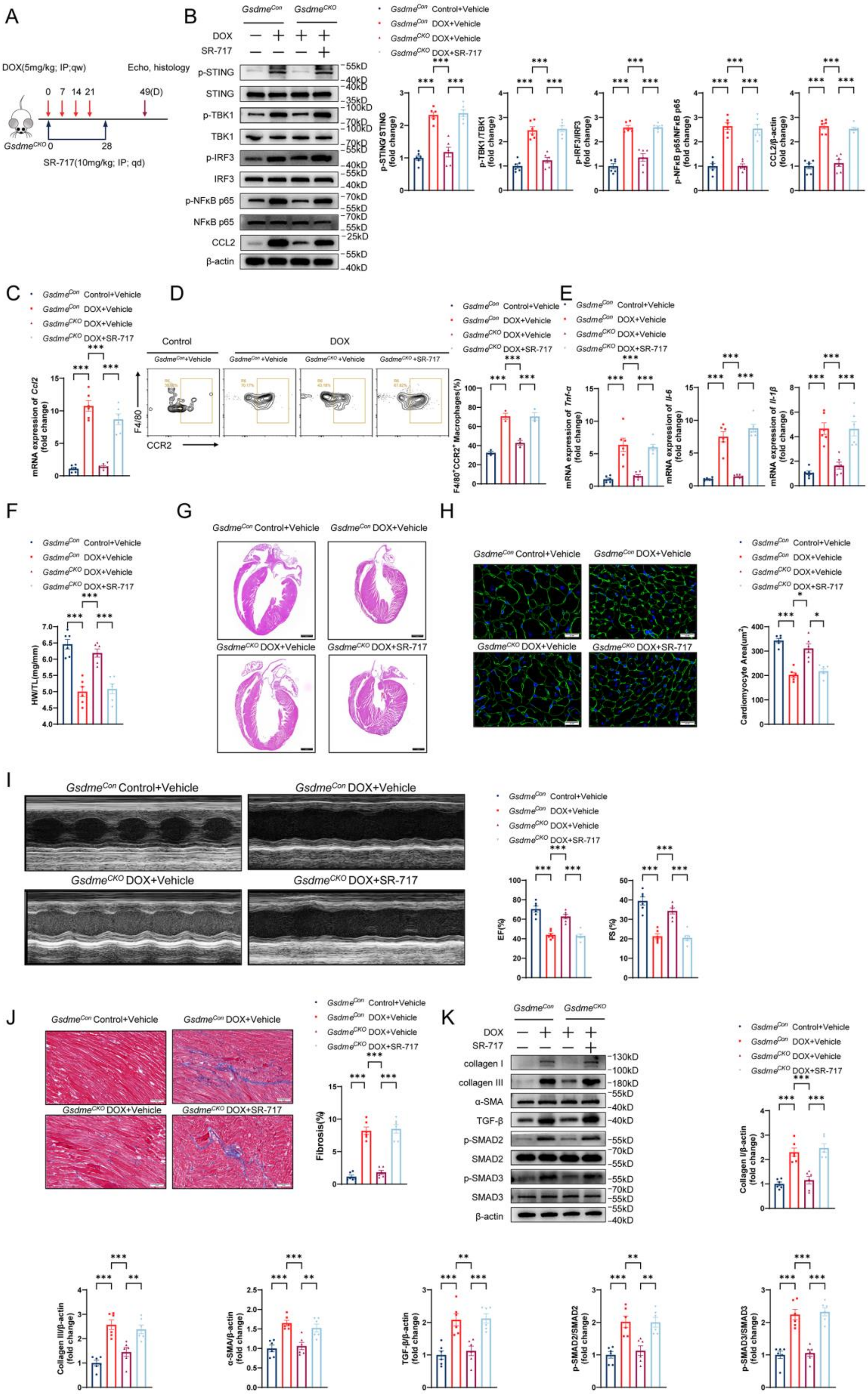
A
DOX(5mg/kg; IP;qw)
Echo, histology
0 7 14 21
49(D)
Gsdme^CKO 0
28
SR-717(10mg/kg; IP; qd)
B
Gsdme^Con
Gsdme^CKO
DOX
SR-717
p-STING
STING
p-TBK1
TBK1
p-IRF3
IRF3
p-NFκB p65
NFκB p65
CCL2
β-actin
Gsdme^Con Control+Vehicle
Gsdme^Con DOX+Vehicle
Gsdme^CKO DOX+Vehicle
Gsdme^CKO DOX+SR-717
p-STING/ STING (fold change)
p-TBK1/TBK1 (fold change)
p-IRF3/IRF3 (fold change)
p-NFκB p65/NFκB p65 (fold change)
CCL2/β-actin (fold change)
C
mRNA expression of Ccl2 (fold change)
D
Control
DOX
F4/80
CCR2
F4/80+CCR2+ Macrophages(%)
E
mRNA expression of Tnf-α (fold change)
mRNA expression of Il-6 (fold change)
mRNA expression of Il-1β (fold change)
F
HW/TL(mg/mm)
G
H
Cardiomyocyte Area(um²)
I
EF(%)
FS(%)
J
Fibrosis(%)
K
collagen I
collagen III
α-SMA
TGF-β
p-SMAD2
SMAD2
p-SMAD3
SMAD3
Collagen I/β-actin (fold change)
Collagen III/β-actin (fold change)
α-SMA/β-actin (fold change)
TGF-β/β-actin (fold change)
p-SMAD2/SMAD2 (fold change)
p-SMAD3/SMAD3 (fold change)

Figure S7. STING agonist SR-717 reverses the cardioprotective role of *Gsdme* knockout in DIC

A, Schematic of SR-717 administration via intraperitoneal injection in DIC *Gsdme*^{CKO} mice. **B,** Representative western blots and gray-band intensities of p-STING/STING, p-TBK1/TBK1, p-IRF3/IRF3, p-NFκB p65/NFκB p65 and CCL2 in the hearts of SR-717-treated DIC *Gsdme*^{CKO} mice; n = 6 per group. **C,** RT–qPCR detection of *Ccl2* transcription levels in the hearts of SR-717-treated DIC *Gsdme*^{CKO} mice; n = 6 per group. **D,** Representative flow cytometry images and quantitative analysis of cardiac CD45+CD11b+F4/80+CCR2+ macrophages; n = 3 per group. **E,** RT-qPCR detection of the mRNA levels of *Tnf-α*, *Il-6* and *Il-1β* in the hearts of SR-717-treated DIC *Gsdme*^{CKO} mice; n = 6 per group. **F,** Ratio of HW to TL in DOX-treated *Gsdme*^{CKO} mice upon SR-717 administration; n = 6 per group. **G,** Representative H&E staining of heart sections from DOX-treated *Gsdme*^{CKO} mice upon SR-717 administration. Scale bar = 1000 μm. **H,** Representative WGA staining and quantitative analysis of the cardiomyocyte cross-sectional area of DOX-treated *Gsdme*^{CKO} murine hearts upon SR-717 administration. Scale bar = 20 μm; n = 6 per group. **I,** Representative echocardiograms and quantification of left ventricular EF and FS in DOX-treated *Gsdme*^{CKO} mice upon SR-717 administration; n = 6 per group. **J,** Representative Masson's trichrome staining and measurement of the myocardial fibrotic area in hearts from DOX-treated *Gsdme*^{CKO} mice upon SR-717 administration. Scale bar = 50 μm; n = 6 per group. **K,** western blot images and quantitative analysis of collagen I, collagen III, α-SMA, TGF-β, phospho-SMAD2, total-SMAD2, phospho-SMAD3, and

total-SMAD3 in hearts from DOX-treated *Gsdme*^{CKO} mice upon SR-717 administration; n = 6 per group. **P < 0.01, ***P < 0.001.

Reference

1. Ackers-Johnson M, Li PY, Holmes AP, O'Brien SM, Pavlovic D, Foo RS. A Simplified, Langendorff-Free Method for Concomitant Isolation of Viable Cardiac Myocytes and Nonmyocytes From the Adult Mouse Heart. Circ Res. 2016; 119: 909-20.
2. Zhang X, Zuo X, Yang B, Li Z, Xue Y, Zhou Y, et al. MicroRNA directly enhances mitochondrial translation during muscle differentiation. Cell. 2014; 158: 607-19.
